# Evolutionary inference reveals global natural histories and predicted pathways of antimicrobial resistance in *Klebsiella pneumoniae*

**DOI:** 10.1371/journal.pbio.3003848

**Published:** 2026-06-12

**Authors:** Olav N. L. Aga, Sabrina J. Moyo, Joel Manyahi, Upendo Kibwana, Iren H. Löhr, Nina Langeland, Bjørn Blomberg, Iain G. Johnston

**Affiliations:** 1 Department of Clinical Science, University of Bergen, Bergen, Norway; 2 Computational Biology Unit, University of Bergen, Bergen, Norway; 3 Department of Medicine, Haukeland University Hospital, Bergen, Norway; 4 Muhimbili University of Health and Allied Sciences, Dar es Salaam, Tanzania; 5 Tropical Disease Biology Department, Liverpool School of Tropical Medicine, Liverpool, United Kingdom; 6 Department of Medical Microbiology, Stavanger University Hospital, Stavanger, Norway; 7 National Advisory Unit for Tropical Infectious Diseases, Haukeland University Hospital, Bergen, Norway; 8 The Norwegian Institute of Public Health, Oslo, Norway; 9 Department of Mathematics, University of Bergen, Bergen, Norway; Wageningen University, NETHERLANDS, KINGDOM OF THE

## Abstract

Antimicrobial resistance (AMR) is a substantial and growing global health burden. Understanding, and predicting, its evolution in specific pathogens will help responses across scales from individual patient cases to large-scale policy. Here, we use global data on AMR features, predicted from 47k *Klebsiella pneumoniae* genomes, with hypercubic transition path sampling to infer the evolutionary pathways by which AMR features in *K. pneumoniae* (KpAMR) are acquired across 102 countries, territories, and areas. We identify “globally consistent” evolutionary behaviors that hold across countries, and “globally divergent” behaviors including carbapenem and fluoroquinolone resistance that vary across countries. We show how these divergent dynamics covary both with public health superregion and drug use policy, and reveal competing evolutionary pathways within and between countries. Using newly sequenced data across several decades from sub-Saharan Africa, we show that this inferred global roadmap of KpAMR evolution successfully predicts prospective evolutionary dynamics. Together, we hope that the ability to characterize and predict evolutionary dynamics of AMR acquisition, connected to socio-economic and drug policy predictors, will help strengthen our understanding of AMR evolution worldwide.

## Abstract 2

Antimicrobial resistance in Klebsiella pneumoniae is a growing global threat, but its evolutionary routes remain hard to predict. This study uses machine learning to explore how resistance evolves across different countries, revealing shared and region-specific pathways that may help forecast resistance evolution.

## Introduction

Antimicrobial resistance (AMR), the resistance of microbial pathogens to the medicines we use to treat them, is a major threat to global health. Antibiotic resistance, the resistance of bacteria to antibacterials, is an important subset of AMR. In 2019 and 2021, 1.27m and 1.14m deaths, respectively, were attributable to bacterial AMR (many more were associated with AMR), with highest rates in sub-Saharan Africa [1,2]. The evolution of genetic and phenotypic features that cause AMR in bacterial pathogens is the focus of tremendous research interest, from basic biology through epidemiology, clinical studies, and health policy.

*Klebsiella pneumoniae* (Kp) is a gram-negative bacterium and a major cause of nosocomial infections across settings. (Related species and subspecies of Kp form the KpSC (species complex); in this report, we will focus on *K. pneumoniae* sensu *stricto*). Kp is found in multiple environmental niches and can both cause opportunistic infections in hospitalized patients and community acquired infections, including severe infections caused by hypervirulent strains [3]. Kp has been identified as an important trafficker for antibiotic resistance genes from different ecological niches into several other clinically important pathogenic bacteria [4]. Kp contributes significantly to the global burden of AMR. In 2021, 45,600 and 36,000 deaths were attributed to carbapenem and fluoroquinolone-resistant Kp, respectively. In 2021, Kp accounted for 12.7% of all deaths attributable to AMR in the population over 5 years old and 19.4% of AMR-attributable deaths in children under five years [2].

Given the substantial health burden associated with the evolution of AMR in Kp, large-scale data-driven research has explored the genomic structure of, and AMR features in, Kp populations [3,5–8]. Here, we consider a complementary perspective: the evolutionary pathways by which these features are acquired [9]. Many related subquestions here can broadly be unified as: from a global perspective, in what orders are AMR features acquired over different instances of pathogen evolution? For example, given a newly observed isolate in the clinic with a given set of AMR features, can we predict which drug resistance(s) will evolve next (and thereby inform treatment guidelines accordingly)? And can we identify which extrinsic features—from physical environment, demography, drug policy, or others—influence the evolutionary acquisition of AMR?

These questions require approaches that can use the expanding volumes of AMR data to make and train descriptive and predictive evolutionary models. The factors shaping evolutionary pathways of AMR are increasingly studied with a combination of modeling and bioinformatic approaches [10], including connections with drug use [11], the role of structure and heterogeneity in bacterial populations [12], and evolutionary connections between AMR features [13]. However, the large sets of potentially coupled features involved in typical AMR systems are often challenging to address using traditional methods from evolutionary biology. For example, an often-studied dataset of *Mycobacterium tuberculosis* isolates contains susceptibility-resistance information for a panel of 10 different drugs [14]. Comparative methods like the Mk (Markov k-state) model often struggle for more than around 7 potentially coupled characters under study [15]. An alternative class of methods for studying the evolution of multiple characters—evolutionary accumulation modeling or EvAM—has emerged jointly from the cancer and evolution literatures [16,17], but has traditionally considered relatively few characters in independent samples, while AMR research questions often involve many different drug resistance features in samples connected by phylogenetic relationships where presence may be due to inheritance from a common ancestor rather than independent acquisitions [9,18,19].

Hypercubic transition path sampling (HyperTraPS) is an EvAM approach designed to incorporate phylogenetic information into the (Bayesian) inference of evolutionary pathways for multiple interacting characters [20–22]. Other EvAM approaches including TreeMHN [23], HyperHMM [24], and HyperMk [15], also support phylogenetically coupled data, but we focus on HyperTraPS here because of its flexibility and support for relatively large numbers of features [20]. In this article, we harness large-scale global datasets on AMR in Kp (KpAMR) with HyperTraPS to learn the structure and variability of evolutionary pathways of drug resistance in Kp across the globe, as well as the extrinsic factors shaping these pathways.

## Results

### Local and global inferred pathways of AMR evolution in Klebsiella

**Fig 1:**
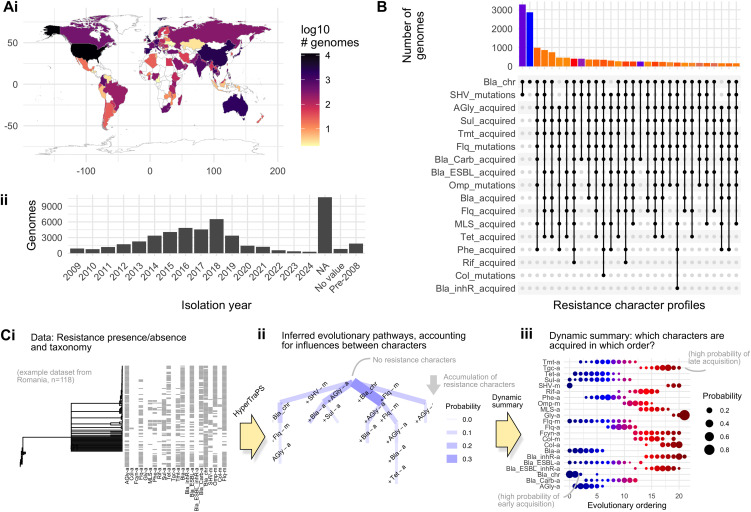
Using global genomic data on KpAMR to learn evolutionary pathways. **(A)** We use 47,721 *Klebsiella pneumoniae* (Kp) isolates from around the world, with (i) country-specific counts of isolates given by color and years of isolation given in (ii). **(B)** UpSet plot giving counts of the 30 most common KpAMR character profiles across these isolates (bar colors give number of acquired characters). **(C)** (i) Example source data from Romania, with 118 Kp isolates connected via a putative phylogeny derived from LIN codes (see Methods) and assigned presence (dark pixel) or absence (white pixel) markers for resistance to each of 22 antibiotic groups. (ii) A subset of the hypercubic transition graph inferred by HyperTraPS for Romanian data. Each edge’s width gives the probability of a transition involving a single character acquisition from the upper state to the lower state. The uppermost state is the ancestral state with no AMR characters. Only early transitions above a threshold probability 0.025 are plotted for clarity. (iii) Summary “bubble plot” of evolutionary pathways inferred for Romanian data with HyperTraPS. The area of a bubble gives the posterior probability that a given character (row) is acquired at a given ordering in the accumulative evolutionary process. For example, *Bla_chr* is inferred to be a likely early acquisition, while *Bla_ESBL-a* likely occurs later and *Tgc_a* is inferred likely later still (or never). Color follows a spectrum from early to late orderings. **Character names**: *AGly* (resistance to aminoglycosides); *Col* (colistin); *Fcyn* (Fosfomycin); *Flq* (Fluoroquinolones); *Gly* (Glycopeptides); *MLS* (Macrolides); *Phe* (Phenicols); *Rif* (Rifampin); *Sul* (Sulfonamides); *Tet* (Tetracyclines); *Tgc* (Tigecycline); *Tmt* (Trimethoprim); *Bla_a* (β-lactam resistance without extended spectrum or inhibitor-resistance); *Bla_inhR* (resistance to β-lactam/inhibitor combinations); *Bla_ESBL* (resistance to extended-spectrum β-lactams); *Bla_ESBL_inhR* (resistance to extended-spectrum β-lactam/inhibitor combinations); *Bla_Carb* (resistance to carbapenems); *SHV* (SHV β-lactamase with expanded enzyme activity), *Bla_chr* (SHV alleles conferring resistance to ampicillin), *Omp* (outer membrane protein). More details available via Kleborate documentation at https://github.com/klebgenomics/Kleborate. We will use *-a*: acquired (via horizontal gene transfer); *-m*: mutation; *-chr*: chromosomal (intrinsic). The map in this figure is from R package *maps* [26] which uses geographical data from Natural Earth as a base layer https://www.naturalearthdata.com/downloads/50m-physical-vectors/ (public domain https://www.naturalearthdata.com/about/terms-of-use/). The data and code underlying this figure can be found at https://doi.org/10.5281/zenodo.20408311.

To learn and compare the likely pathways by which KpAMR is acquired in different countries, we obtained a dataset of 47,721 genomes from 102 different countries, territories and areas, and extracted details of the presence or absence of genes corresponding to each of 22 AMR classes, using Pathogenwatch and Kleborate [5,25] (Methods; Fig 1A and 1B). We refer to presence/absence of resistance genes for each of these classes as binary “resistance characters” (“character” referring to a particular property of a species in evolutionary biology). The set of characters we consider, following Kleborate, is given in the Fig 1 caption; in this report, we will use the shorthand defined there, grouping genes by drug resistance class (and Lahey class for β-lactamases) [25,27,28].

For each country in our dataset, we next used HyperTraPS, a machine learning approach, to infer the “pathways” of KpAMR character acquisition: that is, the *ordered sequences* with which characters are acquired over time (see Methods). Fig 1C illustrates this pipeline for a single country example (Romania, chosen for its moderate number of samples). HyperTraPS takes presence-absence patterns of characters on a phylogeny as input (Fig 1Ci) and outputs an evolutionary model describing the pathways of character acquisition most supported by the data [20–22]. The full output of the inference process is a parameterized transition network describing the probability of different sequences of transitions through the state space of possible resistance characters (Fig 1Cii). This model can be summarized, for example, as the set of posterior probabilities *P*_*ij*_ with which character *i* is acquired at ordering *j* in a putative evolutionary process (Fig 1Ciii). We refer to a plot where *P*_*ij*_ is visualized with point size, as in Fig 1Ciii, a “bubble plot”.

In Methods, we describe some nuances involved in using HyperTraPS with these data. KpAMR characters may be acquired reversibly (for example, via plasmid gain and loss), while the model underlying HyperTraPS pictures acquisitions as irreversible. This irreversibility picture reduces a potentially vast (or infinite) intractable set of possible evolutionary pathways to a tractable set (Methods; [29,30]). This increases the uncertainty of inference and challenges the inference of character *interactions*, but evolutionary *orderings*—which characters are likely present when another is acquired—can generally be robustly inferred (demonstrated in S1A and S1C Fig and more deeply in [31]). The approach does not assume an “end point” of all acquired KpAMR characters exists, that acquisitions occur one at a time, nor that populations are perfectly sampled, homogeneous, or perfectly representative of the labeled country of origin with no mixing [12,32]. The LINcode taxonomy linking observations (see Methods) is used to guard against potential pseudoreplication due to relatedness of samples [9,18,19,33], but in practise (given the deep-branching nature of many relationships) has a negligible effect on inference outputs [31,34] (S1B Fig). Finally, HyperTraPS does not assume the presence, absence, or form of interactions between features—evolutionary dynamics can be reported for a “null model” of independent characters (with prevalence reflecting individual rates) and/or interacting characters (where rates depend on acquired characters) from the same model framework. Our focus is on characterizing likely ordering dynamics, and not directly on the interactions between characters.

**Fig 2:**
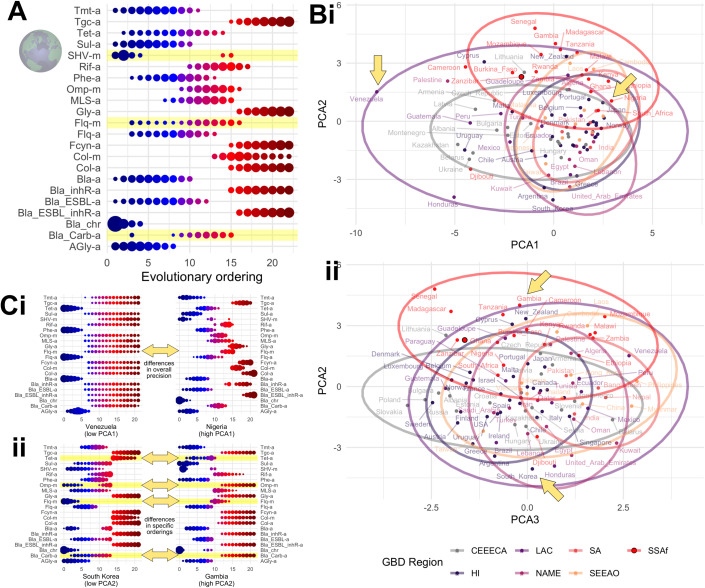
Global structure of inferred pathways of KpAMR evolution. **(A)** Global plot of summarized KpAMR evolutionary dynamics aggregated across countries. Characters displaying bimodal acquisition distributions—characteristic of distinct evolutionary pathways—are highlighted. **(B)** Principal components analysis (PCA) of country-specific “bubble plots” as in Fig 1Ciii, reflecting inferred evolutionary dynamics of KpAMR in different countries. Individual countries are plotted on PCAs 1–2 (i) and 2–3 (ii), individually and with ellipses demarcating their corresponding Global Burden of Disease region ([2]; acronyms below). **(C)** Example “bubble plots” from countries (arrowed in (B)) reflecting the extremes of PCA1 (i) and PCA2 (ii). In (i), Venezuela reflects poorly characterized evolutionary dynamics, with relatively uniform probabilities everywhere; Nigeria reflects precisely characterized evolutionary dynamics, with individual characters having highly likely, precisely specified orderings. In (ii), country-specific differences in orderings emerge, including in those characters highlighted. For example, KpAMR evolution in South Korea is likely to involve *Flq-m* acquisition earlier than other characters; evolution in The Gambia is likely to involve *Flq-m* acquisition later than other characters. GBD regions: CEEECA, Central Europe, Eastern Europe, Central Asia; HI, High-income; LAC, Latin American and Caribbean; NAME, North Africa and Middle East; SA, South Asia; SEEAO, South-east and East Asia and Oceania; SSAf, sub-Saharan Africa. Zanzibar (dark outline) is from data newly sequenced in this study. Character names given in Fig 1 caption. The globe image is public domain from WikiCommons https://commons.wikimedia.org/wiki/File:Globe.svg. The data and code underlying this figure can be found at https://doi.org/10.5281/zenodo.20408311.

While Fig 1C demonstrates our approach for a single country, Fig 2A gives the average acquisition probabilities across all countries in our dataset (including grouping by health region from the Global Burden of Disease initiative [2]), and S2 Fig gives the corresponding summary plots for every country in the dataset. Resistance to some antibiotic groups is consistently observed across all countries: following very common presence of *Bla-chr* and *SHV-*m, resistance characters against betalactams and extended-spectrum betalactams (ESBLs) (*Bla-a*, *Bla_ESBL-a*) are often acquired earlier, while resistance to colistin (drug of last resort for multi-drug resistant K_p_; *Col-a* and *Col-m*) is acquired later. Country-specific differences exist in the evolutionary acquisition of other characters, including resistance to carbapenems (*Bla_Carb-a)*.

In several instances, bimodality in the inferred evolutionary dynamics is observed. That is, a given character may be acquired at an earlier stage or a later stage in the evolutionary process, but not at intermediate stages. Such bimodality is characteristic of mutually repressing interactions between characters, and equivalently of several different evolutionary pathways existing in a system. Examples can readily be seen in Fig 1C for Romania and Fig 2A for the global mean behavior, where *SHV-m* is likely acquired either very early or at more intermediate stages, with little probability in between. These competing dynamics are directly visible in Fig 1Cii, where distinct, canalized pathways exist: one involving *SHV-m* as the first acquisition, one involving *Bla_chr* as the first acquisition and *SHV-m* only following rather later. Across countries (S2 Fig), bimodality is observed in several characters: some examples are in *Bla_chr* (Tanzania, Zambia); *Bla-a* (Ireland, S Korea); *Bla_ESBL-a* (Laos); and *SHV-m* (Romania and at least 15 other countries across GDB regions), reflecting multiple evolution pathways for both HGT-mediated and other KpAMR character acquisitions. Correspondingly, the country-specific inferred orderings of KpAMR characters generally correlate, but rather weakly (R^2^ = 0.41), with the simple prevalence of characters in that country’s source data (S3 Fig).

Across subsets of countries, interactions between our KpAMR resistance characters are observed (S4 Fig). For example, in around a quarter of cases, the acquisition of tigecycline resistance (*Tgc_a*) is inferred to promote the acquisition of glycopeptide resistance (*Gly_a*), and the acquisition of sulfonamide resistance (*Sul_a*) is inferred to promote the acquisition of trimethoprim resistance (*Tmt_a*). This latter observation reflects an independently known connection: *sul* and *dfr* genes providing *Sul_a* and *Tmt_a* resistance, respectively, are often genetically linked, and mechanistically act on connected steps in the folate synthesis pathway [35,36]. In some cases, the acquisition of MLS resistance (*MLS_a*) is inferred to repress the acquisition of carbapenem resistance (*Bla_carb_a*), suggesting possible interactions that could be clinically exploited in combination therapies (see Discussion). However, the inference of interactions between reversibly acquired features is challenging using HyperTraPS [31], so these observations must be regarded with some caution.

While S2 Fig gives a detailed “roadmap” of KpAMR evolution across countries, it is hard to immediately extract global-scale insights from this representation. We therefore next considered how to compare these results in a reduced-dimensionality picture.

### Global consistency in evolutionary acquisition of resistance to a subset of drug families

To proceed, we used principal components analysis (PCA) to embed the high-dimensional set of posterior probabilities in a 3D space (Fig 2B). Here, the axes of highest variance in the inferred evolutionary pathways are identified and used to naturally lay out the individual country outputs. Comparison of country-by-country behavior along these axes can then reveal major sources of variability (and similarity) in inferred KpAMR evolution across countries. We underline that migration means that samples from one country likely represent a mixture of bacteria that have evolved in, and outside, that country, and that perfect separation between intrinsic dynamics of countries is therefore not possible with these data (see Methods).

Inferred evolutionary pathways for Kp across countries consistently involve early acquisition of characters conferring resistance to aminoglycosides, sulfonamides, trimethoprim, and betalactams (*AGly*, *Sul*, *Tmt*, and *Bla* characters). Aminoglycosides and sulfonamides were both discovered and entered clinical use early in the mid 20th century. Resistance properties inferred to be acquired at later orderings include extended-spectrum beta-lactam and phenicol resistance, acquired via mobile genetic elements (*Bla_ESBL-a*, *Phe-a*). Acquisitions inferred to be acquired late across countries include inhR-driven resistance (*Bla_inhR-a*, *Bla_ESBL_inhR-a*), and resistance to later- and final-line drugs including colistin, fosfomycin, tigecyclin, and glycopeptides (*Col-a*, *Fcyn-a*, *Tgc-a*, *Gly-a*). These form a set of “globally consistent” evolutionary observations.

### Country-to-country differences in evolutionary acquisition of resistance to carbapenems, fluoroquinolones, tetracycline, and others

The general patterns above appear across evolutionary dynamics across countries and regions, with the first principal component of inferred Kp variability (accounting for 23% of the overall variance) simply reporting the precision with which these patterns can be characterized (Figs 2Ci and 3). In countries supporting less precise inference (for example, with limited datasets), the expected ordering of these characters is more uniform and closer to the average null hypothesis of all characters behaving equally; in countries with more precise output the expected orderings diverge to early and late values (Figs 2Ci and 3).

**Fig 3:**
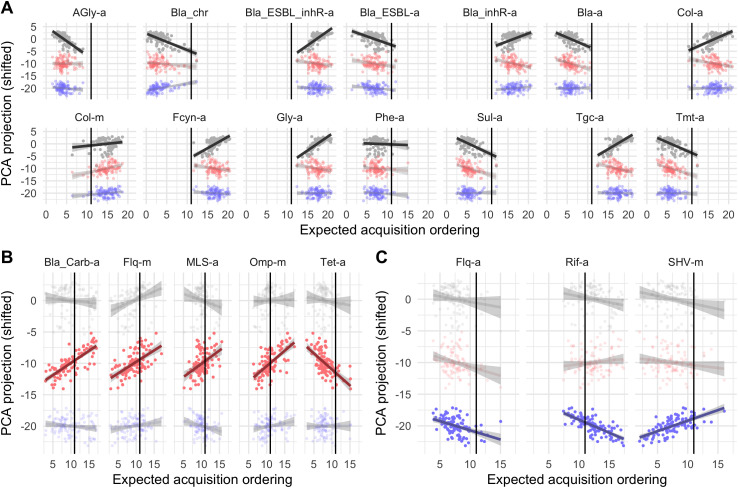
KpAMR characters determining aspects of global variability in evolutionary dynamics. Each country has an expected acquisition ordering for each KpAMR character. The plots show how these patterns of expected ordering are linked to the principal components of global KpAMR evolution variability in Fig 2B: each point plots a country’s expected ordering for a given drug against the country’s position in PCA1 (top, gray); PCA2 (center, red); and PCA3 (bottom, blue). **(A)** “Globally consistent” characters: those that covary tightly with PCA1. All characters here have either early or late expected orderings across all countries (either left or right of the central vertical line). Their position on PCA1 reflects the precision of the inference output. Less precise outputs (low PCA1 values, like Venezuela in Fig 2C) will have orderings close to the central “null”; more precise outputs (high PCA1 values, like Nigeria in Fig 2C) will have more divergent “early” and “late” orderings towards the edges of the plot. **(B, C)** “Globally divergent” characters: those that covary more tightly with PCA2 (B) or PCA3 (C). Expected orderings in these character sets span a wider range, showing sources of variability in the evolutionary dynamics that are independent of PCA1 (and hence less influenced by observational uncertainty). An alternative visualization is given in S5 Fig. Character names given in Fig 1 caption. The data and code underlying this figure can be found at https://doi.org/10.5281/zenodo.20408311.

Once variability in this precision is accounted for (by considering the first principal component), variability corresponding to other differences in the inferred dynamics can be extracted. Several characters covary comparatively little with the (precision-aligned) PCA1 and instead show stronger covariance with PCA2 or PCA3, suggesting that there is genuine country-to-country variability in these characters after controlling for differences in observational noise (Figs 2Cii, 3, and 5). PCA2 (accounting for 15% of overall variance) displays strong covariance with carbapenem, fluoroquinolone, MLS, and tetracycline resistance and OMP mutation (*Bla_Carb-a*, *Flq-m*, *MLS-a*, *Tet-a, Omp-m*,). PCA3 (accounting for 8% of overall variance) displays covariance with *Flq-a*, *Rif-a*, *SHV-m*. None of these “globally divergent” characters display the “null-to-extreme” divergence seen in those characters most closely related to PCA1, suggesting genuine country-to-country differences in evolutionary dynamics, rather than differences in sample size or posterior precision, are responsible for this variability.

**Fig 4:**
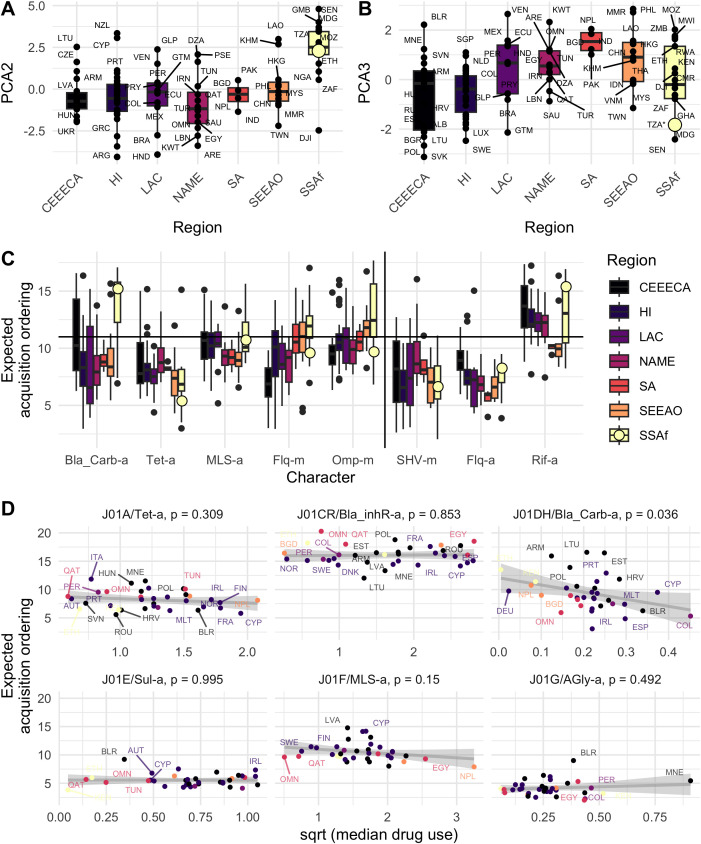
Geographical and drug policy influence on inferred AMR evolutionary pathways. **(A, B)** Distributions of country-specific PCA projection values across different GBD regions for (A) PCA2 and (B) PCA3. In (A), sub-Saharan African countries show systematically higher positions in PCA2 than other regions. **(C)** Inferred acquisition orderings of KpAMR characters by GBD region. The left five characters covary with PCA2; the right three covary with PCA3. Carbapenem resistance and OMP mutations are inferred to be acquired substantially later in sub-Saharan Africa; rifampicin and fluoroquinolone resistance are inferred to be acquired substantially earlier in S, SE, E Asia, and Oceania. New Zanzibar data from this study is shown by large circles. **(D)** Connecting AMR character acquisition propensity with drug treatment regimens across countries. Median (across observed years 2016–2021) per-capita drug use in different drug classes (J codes), with expected acquisition ordering of associated resistance characters. “Globally consistent” characters *Tet-a*, *Bla_inhR-a*, *Sul-a, AGly-a* were associated with PCA1 in the global analysis and display little correlation between evolutionary ordering and drug use levels. “Globally divergent” characters *Bla_Carb-a* and *MLS-a* were associated with PCA2 in the global analysis and display stronger correlations, with earlier evolution of resistance linked to higher levels of drug use. Character names given in Fig 1 caption; *p*-values are from linear regression against a null hypothesis of zero slope. The data and code underlying this figure can be found at https://doi.org/10.5281/zenodo.20408311.

**Fig 5:**
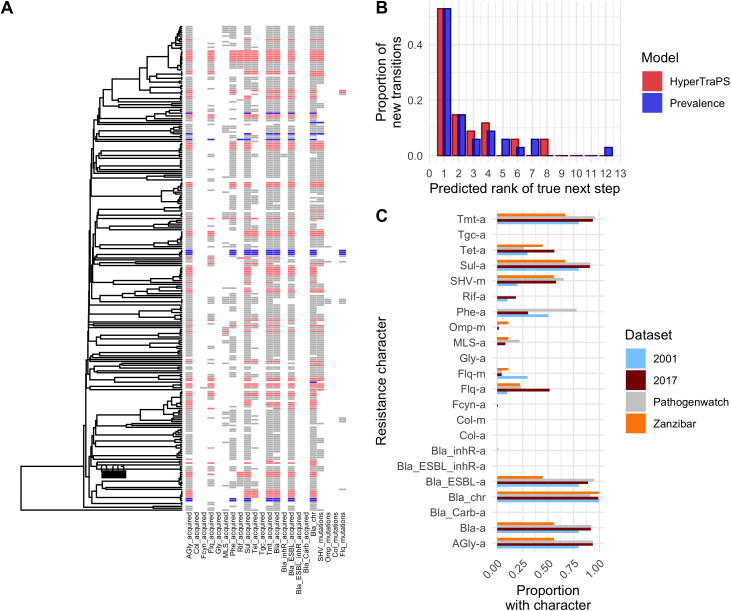
Validating prospective predictions of evolutionary transitions with newly sequenced KpAMR data. **(A)** PathogenWatch (gray) and newly sequenced 2001−2002 (blue) and 2017−2018 (red) genome properties from Tanzania, connected by a phylogeny estimated from average nucleotide identity (ANI). More details in S6 Fig. **(B)** For each independent transition in the set of newly sequenced observations, we used HyperTraPS trained on existing data to predict which characters would likely evolve next given the ancestral state. The bars show the predicted rankings of the characters that were truly observed to evolve in the data. The trained model (red) usually predicted top rankings for the characters that were truly next acquired, marginally outperforming a model based purely on character prevalence (blue). **(C)** Proportion of Tanzanian isolates in Kleborate and new datasets (uncorrected for relatedness) where different KpAMR characters are present. Character names given in Fig 1 caption. The data and code underlying this figure can be found at https://doi.org/10.5281/zenodo.20408311.

### Geographical correlates with Kp evolutionary dynamics

To explore the sources of this evolutionary variability in more detail, we asked whether geographical region was linked to different variants of the inferred evolutionary dynamics (after accounting for observational differences). We first asked whether countries from different regions showed systematic differences in their PCA2 and PCA3 projections—corresponding to different orderings of the “globally divergent” country-specific characters above (Fig 4). We found that sub-Saharan Africa was a dramatic outlier in PCA2, involving notably later acquisition of carbapenem and fluoroquinolone resistance and OMP mutation than the other regions (Fig 4A and 4C). This ordering result agrees with the continuous-time history of treatment in the region, where carbapenem and fluoroquinolone use was established later (especially for children) than in other regions. Other regions were broadly consistent in PCA2 but showed substantial differences in PCA3. These differences correspond mainly to acquired resistance to fluoroquinolone and rifampicin, with substantially earlier inferred acquisition in South-Southeast-East Asia and Oceania, and substantially later in Central and Eastern Europe, Central Asia, and sub-Saharan Africa (Fig 4B and 4C).

### Policy predictors of variance in AMR evolutionary dynamics

We next asked whether known policy of antibiotic use influence the inferred evolutionary dynamics from our analysis. We gathered statistics on drug use from 2016 to 2021 in countries available from the WHO GLASS surveillance programme [37] and looked at correlations between per-capita drug use statistics and the inferred evolutionary dynamics of resistance to those drugs (Fig 4D). We found that for those drug types associated with PCA1 (where variability in inferred dynamics is largely determined by observation uncertainty) there was little correlation between drug use and the ordering of resistance acquisition. However, for carbapenems, associated with PCA2 (more non-observational country-to-country variability), there was a link between drug usage statistics and inferred resistance evolution, with carbepenam resistance (*Bla-Carb-a*) more generally present before other acquisitions in countries with high drug use rates. This observation could arise through increased *Bla-Carb-a* acquisition rate, or (perhaps more likely) a reduced loss rate of the *Bla-Carb-a* feature once acquired (see Methods). The direction of this correlation was also observed in MLS, also associated with PCA2, though this relationship did not show statistical support at the *p* < 0.05 level. This pattern of correlations is consistent with the picture above: acquisition of some (PCA1) KpAMR characters is relatively constant across countries and circumstances, while other (PCA2) characters are more plastic and depend more tightly on country- and region-specific drivers. We note, however, that the GLASS data only cover a subset of years for which bacterial samples exist in our data (Fig 1A) and that KpAMR evolution is not constrained only within these dates (see Methods), so our drug use measure is only an imperfect estimate of the strength of any selective pressure shaping the dynamics.

### Testing predictions of AMR evolutionary dynamics with newly sequenced, cross-decadal data

One motivating application of evolutionary inference to AMR problems is the ability to predict the next steps in evolutionary pathways. A question of potentially direct clinical relevance is: given a clinically observed isolate with a given set of AMR characters, which character(s) will likely evolve next—and can treatment guidelines be adapted accordingly? Previous studies of AMR with HyperTraPS have demonstrated its ability to predict properties of evolutionary transitions in a withheld subset of the overall dataset, following the common training-test machine learning paradigm [20]. Here, we are in the unique position of using independently obtained Kp genome data to test the predictions of the evolutionary models trained above.

We sequenced Kp isolates from patients with bloodstream infections in 2001−2002 and 2017−2018 in Tanzania and 2015−2016 in Zanzibar, collected in previous studies [38–41] (Fig 5; see Methods). Bioinformatic analysis placed these new genomes in a phylogenetic tree with a subset of existing genomes from the training data, including their AMR profiles (Figs 5A and S6 Fig). Using only transitions independent of those in the training data (see Methods), we tested the predictions of the HyperTraPS model trained on data from Tanzania. Specifically, for each new transition, we queried the trained model about likely next steps from the ancestral state, and recorded the predicted ranks of the next steps actually present in the transition (Fig 5B). By far the most common outcome was that the true next step was ranked first (predicted to be most likely). For these data, we considered the number of instances where one model outperformed the other, in the sense of assigning a lower predicted rank (closer to the effective rank-1 “truth”) to a given transition. The trained model outperformed a naïve model (based only on the relative prevalence of KpAMR traits) in a limited subset (6/34 = 18%) of cases, and underperformed in 1/34 = 3% of cases. This is because the newly-observed data showed only limited evidence of higher-order effects (including bimodal ordering distributions and interactions between characters), which HyperTraPS can readily capture (demonstrated in previous applications to AMR data [20,9], but a prevalence-only model cannot (hence the variability in S3 Fig).

This analysis demonstrates that within-country predictions of future evolutionary dynamics are possible given a trained HyperTraPS model. We also asked whether the model could predict evolutionary dynamics at the regional scale. We use Zanzibar as a test case here—a region of Tanzania that was not explicitly represented in the original Kleborate training dataset. Our findings would predict KpAMR in Zanzibar to fall into the sub-Saharan Africa set of behaviors, with high values on PCA2 and associated signatures of late carbapenem resistance and others (Fig 4A). To this end, we applied HyperTraPS inference to newly sequenced isolates from Zanzibar. The resulting inferred dynamics clearly place Zanzibar within the set of sub-Saharan Africa behaviors (Fig 2B), with high values on PCA2 arising from later acquisition of carbapenem resistance, and high values on PCA3 arising from later acquisition of rifampicin resistance (Fig 4A and 4B).

We also explored the representation of different KpAMR characters in the snapshot genome sequences alone, without connecting with evolutionary dynamics. Fig 5C shows the relative prevalence of acquired KpAMR characters in existing and newly-sequenced data, not accounting for phylogenetic relatedness (and without representation of an explicit non-AMR control group). With this sampling, most characters showed an increase from 2001−2002 to 2015−2016, particularly in mobile genetic acquisition of fluoroquinolone resistance, while the relative prevalence of acquired phenicol resistance and fluoroquinolone resistance mutations decreased. Proportions of these characters were in broad agreement with known coarser-grained observations from Tanzania [42,43] and existing Tanzanian genomes from Pathogenwatch, but we observed more acquired rifampicin resistance and lower acquired phenicol resistance than in existing samples. Our samples from Zanzibar showed lower relative proportions of aminoglycoside and β-lactam resistance, and higher prevalence of outer membrane protein mutations.

We have embedded the trained HyperTraPS KpAMR models in an online Shiny app https://stochasticbiology.shinyapps.io/amr-predict/. Here, a country of interest can be specified, and the KpAMR profile of a sample given by specifying presence or absence of each of our characters. The app will then give the inferred probabilities of each next possible character acquisition, as well as a transition graph describing the likely subsequent pathways for up to five acquisitions in the future (S7 Fig).

## Discussion

Powerful data-driven approaches to study the evolution of AMR are emerging in parallel with large genomic datasets [3,5–8]. Most of these studies focus on the current genomic structure of pathogen populations. In this research, we have attempted to take a complementary perspective, using large-scale genome data while focusing on the (inferred) evolutionary dynamics that produce these genomic patterns. We believe that this new way of working has substantial power to support other data-driven approaches, particularly in the prediction of unseen and future evolutionary behaviors of pathogens [9].

The core technology we use, HyperTraPS, is an instance of evolutionary accumulation modeling or EvAM [16,44]. EvAM has previously been used to learn AMR pathways, including in HIV [45] and multidrug resistance in tuberculosis [20,21,24]. One question that has remained is to what extent such results reflect universal, general behaviors, versus a response to the specific selective pressures arising from a given country’s drug use (for example). Our global comparison suggests a form of answer to this question. A subset of the KpAMR characters we consider appear to behave similarly across countries regardless of specific selective pressure, drug regimes, and other differences (those aligned with PCA1). Another set of characters (those aligned with PCA2–3) evolves in a way that is more dependent on country- and region-specific influences, which we have shown include public health superregions and drug use levels—although certainly other factors, from population change to the prevalence of other diseases, will also influence these dynamics [10,11]. While these results are specific to K_p_, and the characters we consider, this combination of global and country-specific behaviors may reasonably hold across other pathogens.

Inferring evolutionary dynamics of multiple, coupled traits across groups is a challenging problem. There will be countless, multiscale selective influences that impact the evolution of specific lineages of K_p_ around the globe. Our perspective attempts to “coarse-grain” away from these (unobservable) specific pressures and consider their aggregated effect on large-scale genomic properties in populations. This perspective is not without difficulties. In Methods, we describe in more depth several resolutions to challenges including reversibility of KpAMR acquisitions (for example, via plasmid gain and loss), phylogenetic relatedness, mixing between countries, imperfect observations, and presence or absence of interactions between characters. As described there and studied in [31], evolutionary orderings from HyperTraPS inference are rather robust to these issues in real data, which tend to increase uncertainty rather than systematically bias the outputs of inference. The “controlling” for uncertainty via PCA1 in our analysis is therefore expected to ameliorate the effects of these issues. As shown in S1C Fig, the inference of *which characters are likely present when another is acquired* is congruent across our method and an alternative that, while limited in scale, can directly allow for reversibility [15].

In learning estimates for evolutionary dynamics, HyperTraPS attempts to infer any potential interactions between characters—for example, demonstrating an independently known positive connection between sulfonamide and trimethoprim resistance [35,36]. Negative interactions, where the acquisition of one character makes acquiring another character less likely, could (if truly present) form the basis of a combination therapy XY, where resistance to drug X makes resistance to drug Y less likely. These interactions correspond to the phenomenon of *collateral sensitivity* in AMR, where resistance to one drug induces sensitivity to another [46,47]: for example, in *Escherichia coli*, beta-lactamase expression is linked to heightened sensitivity to colistin (among others) [48]. While not observed consistently throughout all countries in our dataset, a subset of countries showed statistical support for some such “repressive interactions” (S4 Fig). These include, for example, MLS resistance repressing the acquisition of carbapenem resistance. However, this is one aspect of inference which *is* challenged when a reversible process is modeled irreversibly [31], and such indications can only suggest more detailed followup investigation to verify and explore mechanisms behind these possibly exploitable collateral interactions.

In conclusion, we hope to show that evolutionary accumulation modeling—inferring the historic evolutionary pathways of AMR acquisition, in addition to considering contemporary properties—can complement and enhance established ways of working in AMR genomic analysis. By considering the dynamics by which global KpAMR patterns have become established, a collection of research directions are opened or expanded, including the similarities and differences between countries, links with social and environmental covariates, and predictions of future dynamics. We anticipate that these methods may readily be applied to provide insight into AMR evolution in other pathogens in future.

## Methods

### Existing *Klebsiella* data acquisition

We obtained all 47,721 *Klebsiella pneumoniae* sensu *stricto* records from Pathogenwatch as of March 2024 [5]. The most common isolation years were 2015–2020, with a peak at 2018 (Fig 1A). We used AMR features (genes and mutations) reported by drug class as reported by Kleborate version 2.3 [25]. Kleborate groups genes or mutations known to confer resistance to clinically relevant antibiotic groups and related phenotypes. Betalactamases are further grouped by enzyme activity [25]. We consider a set of *L* = 22 drug classes for each genome, dichotomizing Kleborate output by the presence of any gene or mutation related to each group. That is, if there is any gene or mutation present for a resistance phenotype, the isolate is considered resistant, otherwise it is considered susceptible. This assumption holds for most resistance phenotypes considered in this study. However, fluoroquinolone resistance (*Flq-m*) often requires multiple mutations to cause resistance. Thus, the presence of these characters cannot be interpreted as a resistance phenotype, but rather an increased MIC (minimum inhibitory concentration). The isolates were divided by country based on metadata, and countries were grouped into superregions as defined by the Global Burden of Disease regional classification system [49]. A coarse-grained phylogeny was generated via LIN-codes (Life Identification Numbers: a systematic, stable quantitative system for genomic classification based on nucleotide identities [50], pre-computed for our dataset [51]) using lincoding.py supplied by [51]. 2,102 genomes were excluded due to missing metadata. Any country with less than two genomes available were excluded due to the inability to construct a tree. The resistance profiles and phylogenetic tree were combined into a phylogenetic tree annotated with resistance to drug classes.

### Evolutionary pathway inference with hypercubic transition path sampling (HyperTraPS)

HyperTraPS [20] models an “evolutionary space” containing every possible state of a system involving *L* binary characters, then infers the probabilities of (Markovian) transitions between states in this space (here, involving the ordered accumulation of different AMR characters) that is most compatible with observed data [21,22]. A recent overview of the method in an AMR context is given in. The source data is a collection of length-*L* “barcodes” labeling presence or absence of our characters—resistance to drug classes as defined in Kleborate—on the tips of a phylogeny. Ancestral state reconstruction (here, assuming that AMR resistance character accumulation is rare and irreversible, so that an ancestor possesses a character if and only if all descendants possess it) is by default used to infer ancestral states, and thereby construct a set of transitions from the data. This process is used solely to guard against pseudoreplication, and for these (largely deeply-branching) datasets, the inclusion of phylogenetic detail (and hence the details of ancestral state reconstruction) has a negligible influence on the inference outputs (S1B Fig). The resulting transitions are the input data for the HyperTraPS algorithm. The target of inference is an *L* × *L* matrix θ, where $\theta_{ii}$ is the base rate for acquiring character *i*, and $\theta_{ij}$ is the influence that acquisition of character *j* has on the base rate of acquisition of character *i* (as in mutual hazard networks [52]). Specifically, the probability of acquiring character *i* from the state corresponding to binary vector *s* is $P(s\text{ gains }i)=\text{exp}(\theta_{ii}+\;\sum {s_{j}\;\theta }_{ij})/\sum_{k}\text{exp}(\theta_{kk}+\;\sum {s_{j}\;\theta }_{kj})$, where $\sum_{k}\;$ is over all characters absent in *s* [20]. In this way, positive and negative interactions between the acquisition of different AMR characters is supported, so that (for example) acquisition of resistance to drug X can make acquisition of resistance to drug Y more (or less) likely. The output of the inference process is, more broadly, a transition matrix λ, with elements $\lambda_{a\to b}$ describing the probability with which an isolate in state *a* will next tr*a*nsition to state *b*. For each of the 102 countries, territories and areas we ran HyperTraPS on three different random seeds to ensure that the results were consistent across runs (S2 Fig). Simulations were run for 10^6^ steps with 10^3^ random walkers for sampling, employing a penalized likelihood involving the HyperTraPS-estimated likelihood for a given set of transitions given a parameterization θ, computed with the probability term above calculated over sampled paths, and a unit penalty for each non-zero parameter (see [20]). The US genomes were sub-sampled to a subset of 1,000 genomes; three different subsamples clustered closer together than with any other countries in the PCA plot.

### Synthetic control study for inference of reversible characters

To determine the effect of our assumption of irreversible accumulation of AMR characters (given that the loss of plasmids is not uncommon in Kp evolution [7]), we constructed a synthetic control study. A random phylogeny over 128 tips was constructed using a birth-death process with birth rate 1 and death rate 0.5 (chosen empirically to roughly match the phylogenies from our case studies) using *phangorn* [53]. A synthetic accumulation process reflecting a single evolutionary pathway was simulated on this phylogeny. Different loss processes—including uniform rate over a subset of features, heterogeneous, and random rates—were simulated in different instances (details in S1A Fig caption). The same HyperTraPS pipeline (assuming irreversibility), including ancestral state reconstruction and pathway inference, was run on both the pre-loss and post-loss datasets, and the outputs compared (S1A Fig). We also applied HyperMk [15], an EvAM approach supporting reversible dynamics, to a reduced subset of the KpAMR data (HyperMk is limited in the scale of data it can analyze), showing both a tight agreement with the HyperTraPS output and that model selection criteria favor the irreversible model (S1C Fig). These are a small-scale illustrative examples; the inference of reversible dynamics under an irreversible model is the focus of study in [31].

### Global structure in inferred pathways

The outputs of HyperTraPS can be summarized as a matrix *P*, where element *P*_*ij*_ gives the posterior probability that character *i* is acquired at ordering *j* in a putative evolutionary process from an ancestor with no AMR characters towards a final state with all AMR characters. For example, *P*_32_ gives the probability that the third character is the second to be acquired in such an evolutionary process. We obtained this matrix for each country in our dataset, then performed PCA on the set of matrices [54]. We observed the structure of matrices with “bubble plots”, where an array of points is plotted with areas proportional to *P*_*ij*_.

### Drug usage data

We collected antibiotic consumption data from 57 countries, territories and areas enrolled in WHO Glass surveillance program in the period 2016–2021 [37]. Overlaying the consumption data with the countries that had 3 or more available genomes reduced the number to 53. We use the median consumption statistics over the available time points for each country.

### New *Klebsiella* data acquisition

The 79 newly sequenced Kp isolates in this article are derived from a collection of studies performed in Tanzania and Zanzibar over the last three decades. These comprise bacterial isolates from (a) blood samples from pediatric patients with bloodstream infections in Dar-es-Salaam, Tanzania in 2001–2002; (b) blood samples from adult patients with bloodstream infections in Dar-es-Salaam, Tanzania in 2017–2018; (c) fecal samples from the patients in (b); and (d) blood samples from adult and pediatric patients with bloodstream infections in Mnazi Mmoja hospital, Zanzibar in 2015–2016. These cultures were sequenced by MicrobesNG (MicrobesNG, Birmingham, UK) using Illumina HiSeq technology. Assembled contigs were provided by the sequencing service, which formed the initial step for the bioinformatics pipeline below.

### New *Klebsiella* data analysis

The analysis pipeline begins with the contigs from the initial sequencing and assembly process. We first used *dnadiff*, which wraps *nucmer* from the MUMmer 3.0 package [55], to scaffold the contigs from a given isolate on the *Klebsiella* reference genome GCA_000240185.2_ASM24018v2 [56] and report statistics of the alignment. We retained only isolates with <20% unaligned bases as instances of *Klebsiella.* Combining the newly sequenced isolates with the existing Tanzanian genomes from Pathogenwatch, we then ran pairwise *dnadiff* across the combined set. We extracted the average identity across the aligned regions for each pair and recorded this as the ANI (average nucleotide identity). We then used *d* = 1 − ANI as a distance measure for phylogenetic tree estimation using the unweighted pair group method with arithmetic mean (UPGMA) [57] implemented in the *phangorn* [53] package in R (R Core 58]. We confirmed that an alternative method, neighborhood joining [59], did not give qualitatively different results for the predictions of AMR evolution. We used AMR profiles of existing and new isolates to reconstruct ancestral states across this complete tree, then identified the set of subtrees that had only newly-sequenced isolates at their tips. As transitions within such subtrees are independent of transitions in the (Pathogenwatch) training data set, we used this set of transitions as our independent test set.

### Testing predicted dynamics from the trained model on new data

We queried the model fitted from the training data to predict the likely next states from each precursor state in this independent set of transitions [20]. We ranked the possible next steps from each state from most likely to least likely under the fitted model. We then recorded the ranks corresponding to the actual step(s) corresponding to the observed transition, and compared these to a null model where each possible transition could occur with probability given by its relative prevalence in the training data. For the Shiny app in S7 Fig, we report the probabilities of each possible next acquisition step given a provided current state, and construct the transition network starting from that current state and involving up to five further acquisitions.

### Notes on nuances and resolutions in applying HyperTraPS to KpAMR data

The model underlying HyperTraPS pictures acquisitions as occurring irreversibly, while KpAMR characters—particularly those acquired through plasmid exchange—may be gained and lost many times by a lineage [9]. The fundamental reason we use an irreversible model is computational tractability. If reversible transitions are allowed, a potentially infinite set of evolutionary pathways (involving repeated acquisition and loss of characters) can connect any two states, complicating the computation of these pathways and their interpretation. In practise—including in AMR systems—only a limited number of the vast space of possible pathways play important roles in evolutionary dynamics [29,30], and powerful results have been demonstrated for accessibility of pathways sets on hypercubic evolution landscapes [60,61].

While applying irreversible models to systems involving reversibility increases the uncertainty of the inference process and challenges the inference of the detailed interactions between features, inferred evolutionary dynamics and particularly acquisition orderings is robust to this challenge—illustrated with some simple examples in S1A Fig, comparison with an EvAM approach allowing for reversibility [15] in S1C Fig, and the focus of study in [31]. This holds both for comparable loss rates across features and for distinct loss propensities, in which case the approach functions to infer the gain/loss rate ratio—which itself determines evolutionary orderings and prevalence patterns [31]. Generally, the outputs of HyperTraPS infer and report *which features are likely present when a given feature is gained*—a quantity that remains well-defined across irreversible and reversible cases. The congruence of this inference across reversible and irreversible approaches can be seen, for example, in S1C Fig.

We also note that the model does not assume that a final “end point” with all characters present is ever reached (or indeed possible), instead leaving uncertain all acquisitions that are not observed in real data. HyperTraPS invokes a “emission” picture, comparable to a hidden Markov model, to connect with observations [22], and therefore does not require complete information or homogeneous populations—it can naturally account for limited sampling of a broader, potentially heterogeneous population [32].

HyperTraPS does not *a priori* assume that the inferred evolutionary dynamics are a consequence of independent character accumulation (where each character’s observed prevalence is determined by a single acquisition rate) or interactions between characters (where the acquisition of a character is influenced by the presence of others) [31]. The “null model” of independence is challenged, for example, by instances of bimodality in inferred orderings – where a character may be acquired before many others or after many others, but not at intermediate stages. But the inferred evolutionary trajectories may be reported and analyzed regardless of which generative model is most supported. (We note, in parallel to this point, that simultaneous acquisition of multiple features is very compatible with our approach; the resulting output simply places equal probabilities over all compatible ordering pathways.)

Our samples are connected by a phylogeny, which we estimate with a taxonomy constructed from LINcodes (see Methods). Accounting for this phylogeny allows us to control against pseudoreplication, where individuals inheriting characters from a common ancestor are interpreted as independent acquisition instances of those characters [9,18,19,33]. Our approach uses the *relative* evolutionary timings from the source data phylogeny (for example, Fig 1Ci) to report the *orderings* of events: which acquisitions precede or follow which others, given that characters may influence each other, with the timescale of these events (see Discussion). In our case, much of the taxonomy corresponds to LINcode elements reflecting a deep timescale, with only separations within the final clusters corresponding to separation timescales under 1,000 years (following a back-of-the-envelope calculation in footnote below). For many KpAMR features, a picture of independent acquisitions is then not unreasonable, as many KpAMR acquisitions may have occurred on a shorter timescale (that of modern human medicine). However, our phylogenetically-embedded approach only biases the inference in the case of strong connection between acquisition patterns and sampling [31], otherwise simply increasing the uncertainty of the inferences [31,34]. In many real-world EvAM cases, whether or not phylogeny is included has an effect on uncertainty but no detectable impact on estimated orderings—demonstrated for two countries in our dataset in S1B Fig. As we control for the different uncertainties across countries in the followup PCA analysis, we use this approach to correct for potential instances of pseudoreplication.

The large majority of genomes with timing information were isolated in the period 2015–2020 (Fig 1A). Our approach here does not directly consider the specific, real-world timings of the evolutionary transitions involved. The precise timescales of the evolutionary process are in principle addressable using a version of our approach called HyperTraPS-CT (continuous time) [20], but this requires a consistent estimation of divergence times between all isolates in the dataset. Importantly, our approach here does not completely neglect a timescale—it is implicit in the phylogenetic relationships included in the source data, and this inclusion both accounts for the temporal ordering of events and “pseudoreplication” as above. However, this approach based on relative evolutionary timings means that we cannot directly tie differences in inferred evolutionary behavior to known real-world historical events shaping AMR profiles: shifts in drug policy, for example, or outbreaks or other events changing the effective size of the evolving population. While our results, focusing on the ordering of acquisition events, already demonstrate descriptive and predictive power in KpAMR evolution across countries, a continuous time picture on a smaller scale in the future (perhaps a single country or small set) would enable finer-grained linking between model prediction and the real-world details of policy and disease history.

Our samples are labeled by country of collection, which we treat as a covariate in our analysis. However, of course, bacteria and their hosts are not physically limited to remain within a given country. The phylogeography of Kp has been studied in depth, for example, in [6,62,63] and certainly involves mixing of strains across borders. Our analysis and interpretation do not assume that observations from a country all and exclusively reflect evolution in that country alone; our regional and correlative downstream analysis accepts that mixing can readily take place and attempts to identify variability that can be observed despite this mixing (which, in the limiting case, would lead to homogeneous inferred dynamics worldwide).

The terminal clusters in our LIN-derived taxonomy reflect ANI > 99.84%, giving a per-site substitution proportion of at most 0.0016. Estimates for Kp substitution rates are around 1.5 × 10^−6^ substitutions per site per year [64]. A substitution proportion of 0.0016 might then be expected over a timescale of 0.0016/1.5 × 10^−6^ ≈ 10^3^ years, reflecting an estimate for the longest timescale of separation within the terminal clusters.

### Data and code availability

The code base for this study is publically available at https://github.com/StochasticBiology/kp-evolution-inference. A Zenodo repository including this codebase, raw FASTA files for the newly-sequenced Kp genomes, and cached versions of the raw data for all figures is available at https://doi.org/10.5281/zenodo.20408311. In addition to the software referenced above, we use R with libraries *hypertrapsct* for evolutionary inference (Aga et al., 2024), *dplyr* (Wickham et al., 2023), *tidyverse* (Wickham et al., 2019), *countrycode* (Arel-Bundock et al., 2018) for data manipulation, *lme4* (Bates et al., 2015) for statistics, *phytools* (Revell, 2012) for phylogenetic analysis, and *ggplot2* (Wickham, 2016), *ggpubr* (Kassambara, 2020), *ggbeeswarm*, *ggrepel* (Slowikowski, 2021), *ggupset* (Ahlmann-Eltze, 2025) for visualisation [65–75]. We also use a Python script for LINcoding by Melanie Hennart, which uses *numpy (Harris et al., 2020)*. The world map in Fig. 1 is from the *maps* package (Becker *et al.*, 2018), itself using geographical data from Natural Earth as a base layer which is public domain https://www.naturalearthdata.com/about/terms-of-use /. The globe image in Fig. 2 is public domain from WikiCommons https://commons.wikimedia.org/wiki/File:Globe.svg.

## Supporting information

S1 Fig Limited issues with modeling reversible dynamics. Plots involve a dataset (left) and “bubble plot” summary of inference as in the text (right). **(A) HyperTraPS recovers accumulation orderings from reversible processes.** Synthetic datasets involving the progressive accumulation, and loss, of characters X1 to X10, with different accumulation and loss rates (i)–(vi). For (i) accumulation rate = 1 and no loss take place. For (ii)–(vi) accumulation rate = 2 and (ii) loss rate is 0.1 for features 1–5 and 0 otherwise; (iii) loss rate increases linearly from 0.05 for feature 1 to 0.5 for feature 10; (iv) loss rate increases linearly from 0.2 for feature 1–2 for feature 10; (v) loss rate for each feature is drawn from *U*(0, 0.5); (vi) loss rate for each feature is drawn from *U*(0, 1). The output inferred from HyperTraPS accurately reports the accumulation ordering of characters, with differences in uncertainty, and ambiguity about characters which are not observed across all samples. This small-scale case study reflects a specific instance of the picture explored more generally in [31]. In all cases, inferred ordering can be interpreted as reporting which features are present when another is gained (see Methods). **(B) Limited impact of neglecting phylogenies.** Datasets from (i) Romania; (ii) Senegal. Inferences shown ignoring phylogeny (cross-sectional picture) vs. including phylogenetic information for ancestral state reconstruction and considering transitions down lineages. Here (and generally), many samples are related only via deep branches and are effectively independent; more recent relatedness within clades has a marginal effect on the uncertainty of posteriors but no noticeable effect on inferred orderings, in agreement with [34,31]. **(C) Agreement of irreversible and reversible inference.** (i) A reduced subset of the full KpAMR dataset, involving 5 features chosen to reflect a spread of prevalences: *Bla_ESBL_a* (1), *Sul_a* (2), *Bla_chr* (3), *Tet_a* (4), *Rif_a* (5). With this reduced set, the HyperMk approach [15] can be used to perform inference allowing for reversibility. Both the bubble plot summary (ii) and the inferred transition network (iii) for HyperMk (“Rev”) are very similar to the HyperTraPS outputs assuming an irreversible picture (“Irrev”), with HyperMk supporting reversible versions of all the same transitions identified (also observed in [31]); the Akaike Information Criterion favors the irreversible picture (393 vs. 553). The data and code underlying this figure can be found at https://doi.org/10.5281/zenodo.20408311.
(TIF)

S2 Fig All posterior orderings of AMR character acquisitions. This “global roadmap” summarizes “bubble plots” describing inferred evolutionary dynamics of KpAMR characters in different countries. Each row is a country, ordered vertically by descending number of Kp samples. Each column is a KpAMR character; the horizontal axis within columns gives evolutionary orderings (from early to late). The size of a point gives the probability that that character is acquired at that ordering in that country. For example, across the vast majority of datasets (including the top row, USA, for example), *Bla_chr* has a high probability of early acquisition and *Gly_acquired* has a high probability of late acquisition. In Cameroon, all characters have similar inferred evolutionary orderings, reflecting the limited data available to make more precise statements. Character names given in Fig 1 caption. The data and code underlying this figure can be found at https://doi.org/10.5281/zenodo.20408311.
(TIF)

S3 Fig Mean posterior orderings and country-specific prevalences of AMR character acquisitions. **(A)** All KpAMR characters with a prevalence >5% and <95% together (*R*^2^ = 0.41). **(B)** Individual KpAMR characters. The data and code underlying this figure can be found at https://doi.org/10.5281/zenodo.20408311.
(TIF)

S4 Fig Inferred interactions between KpAMR characters. An arrow from character X to character Y reports an inferred interaction between the evolutionary acquisition of those characters. Blue arrow: acquiring X makes acquiring Y more likely (“promoting”). Red arrow: acquiring X makes acquiring Y less likely (“repressing”). The number on each arrow gives the number of countries in which that interaction was inferred; only interactions appearing in >15% of countries with a posterior coefficient of variation under 1 are shown. Character names given in Fig 1 caption. However, as the inference of character interactions with HyperTraPS is challenging for reversible systems [31], these results should be treated with caution. The data and code underlying this figure can be found at https://doi.org/10.5281/zenodo.20408311.
(TIF)

S5 Fig KpAMR characters determining aspects of global variability in evolutionary dynamics (alternative visualization). Connected to Fig 3. KpAMR characters (horizontal axis) are plotted by their expected acquisition ordering (vertical axis) for each country (points). The country’s projection on PCA1, PCA2, and PCA3, for those characters that most strongly covary with each PCA axis, are given by the color of a point. As in Fig 3, those characters that covary with PCA1 form a consistent spectrum linked to precision: low values of PCA1 correspond to less precise, more uniform timing, while higher values lead to more precise earlier or later timings. Characters covarying with the other PCAs display wider ranges connected to PCA1-independent variability. Character names given in Fig 1 caption. The data and code underlying this figure can be found at https://doi.org/10.5281/zenodo.20408311.
(TIF)

S6 Fig New KpAMR data from Zanzibar and Dar es Salaam, Tanzania. Phylogenies and KpAMR profiles from new datasets: **(A)** Zanzibar 2015−6. **(B)** Tanzania 2001−2. **(C)** Tanzania 2017−8. **(D)** Pathogenwatch Tanzania. **(E)** Circular projection of the phylogeny of Tanzanian isolates in Fig 5A. Colors: green, 2001−2; blue, 2017−8; red, existing Pathogenwatch data. The data and code underlying this figure can be found at https://doi.org/10.5281/zenodo.20408311.
(TIF)

S7 Fig Shiny app for predictions of future KpAMR character acquisitions. A country of interest is specified, and the KpAMR profile of a sample given by specifying presence or absence of each of our characters. The graphical outputs then give the inferred probabilities of each next possible character acquisition (which can be downloaded in CSV format), as well as a transition graph describing the likely subsequent pathways for up to five acquisitions in the future. Available at https://stochasticbiology.shinyapps.io/amr-predict/.
(TIF)

## Abbreviations

AMR: antimicrobial resistance
ESBLs: extended-spectrum betalactams
HyperTraPS: hypercubic transition path sampling
Kp: Klebsiella pneumoniae
KpAMR: AMR in Kp
PCA: principal components analysis
