# Supplementary figures and images for: Evolutionary inference reveals global natural histories and predicted pathways of antimicrobial resistance in Klebsiella pneumoniae
Source: PLoS Biol. 2026 Jun 12;24(6):e3003848. doi: 10.1371/journal.pbio.3003848 (PMC13278573; doi:10.1371/journal.pbio.3003848)

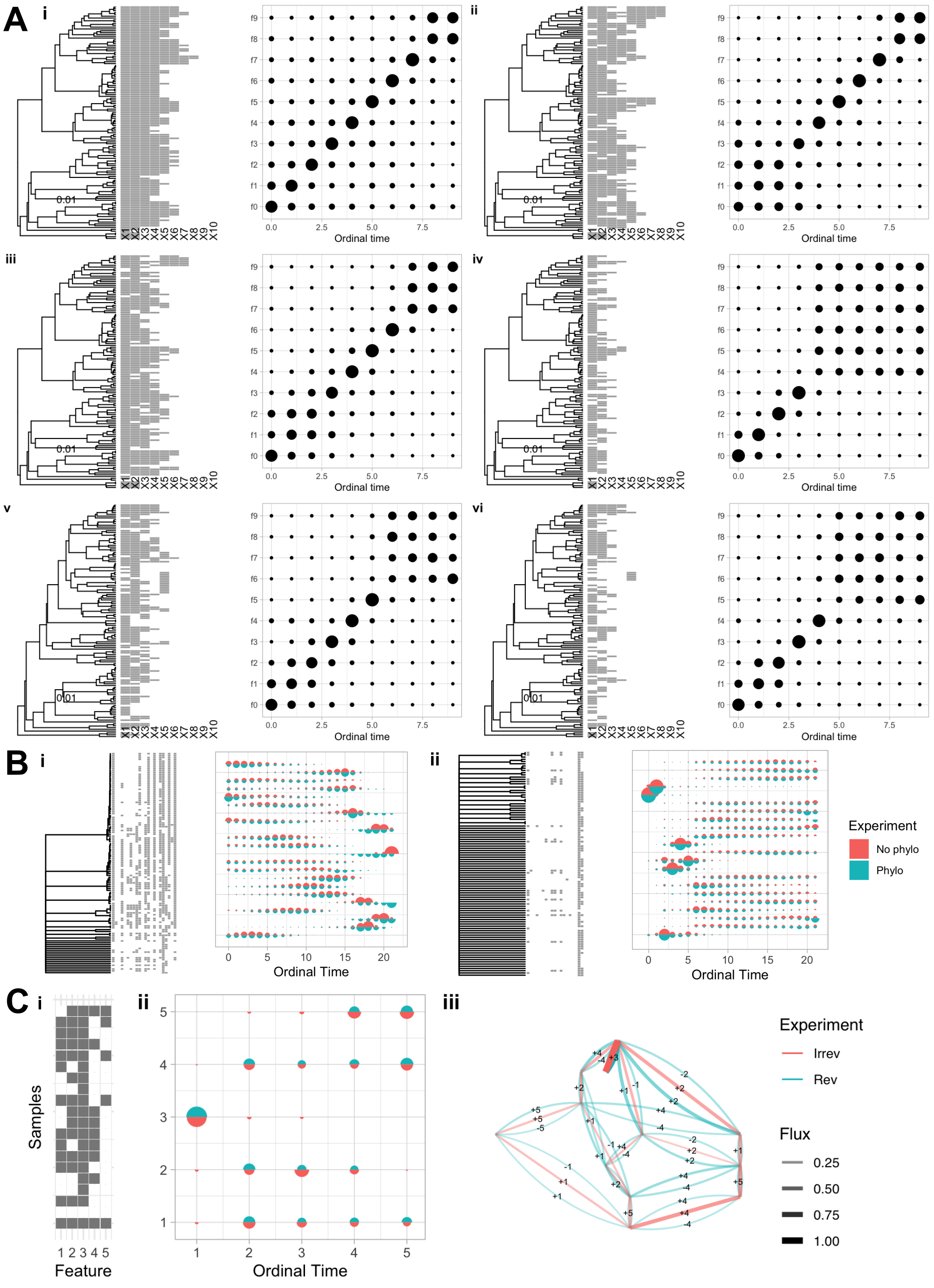

Supplement: S1 Fig — Plots involve a dataset (left) and “bubble plot” summary of inference as in the text (right). (A) HyperTraPS recovers accumulation orderings from reversible processes. Synthetic datasets involving the progressive accumulation, and loss, of characters X1 to X10, with different accumulation and loss rates (i)–(vi). For (i) accumulation rate = 1 and no loss take place. For (ii)–(vi) accumulation rate = 2 and (ii) loss rate is 0.1 for features 1–5 and 0 otherwise; (iii) loss rate increases linearly from 0.05 for feature 1 to 0.5 for feature 10; (iv) loss rate increases linearly from 0.2 for feature 1–2 for feature 10; (v) loss rate for each feature is drawn from U(0, 0.5); (vi) loss rate for each feature is drawn from U(0, 1). The output inferred from HyperTraPS accurately reports the accumulation ordering of characters, with differences in uncertainty, and ambiguity about characters which are not observed across all samples. This small-scale case study reflects a specific instance of the picture explored more generally in [31]. In all cases, inferred ordering can be interpreted as reporting which features are present when another is gained (see Methods). (B) Limited impact of neglecting phylogenies. Datasets from (i) Romania; (ii) Senegal. Inferences shown ignoring phylogeny (cross-sectional picture) vs. including phylogenetic information for ancestral state reconstruction and considering transitions down lineages. Here (and generally), many samples are related only via deep branches and are effectively independent; more recent relatedness within clades has a marginal effect on the uncertainty of posteriors but no noticeable effect on inferred orderings, in agreement with [34,31]. (C) Agreement of irreversible and reversible inference. (i) A reduced subset of the full KpAMR dataset, involving 5 features chosen to reflect a spread of prevalences: Bla_ESBL_a (1), Sul_a (2), Bla_chr (3), Tet_a (4), Rif_a (5). With this reduced set, the HyperMk approach [15] can be used to [file pbio.3003848.s001.tif]

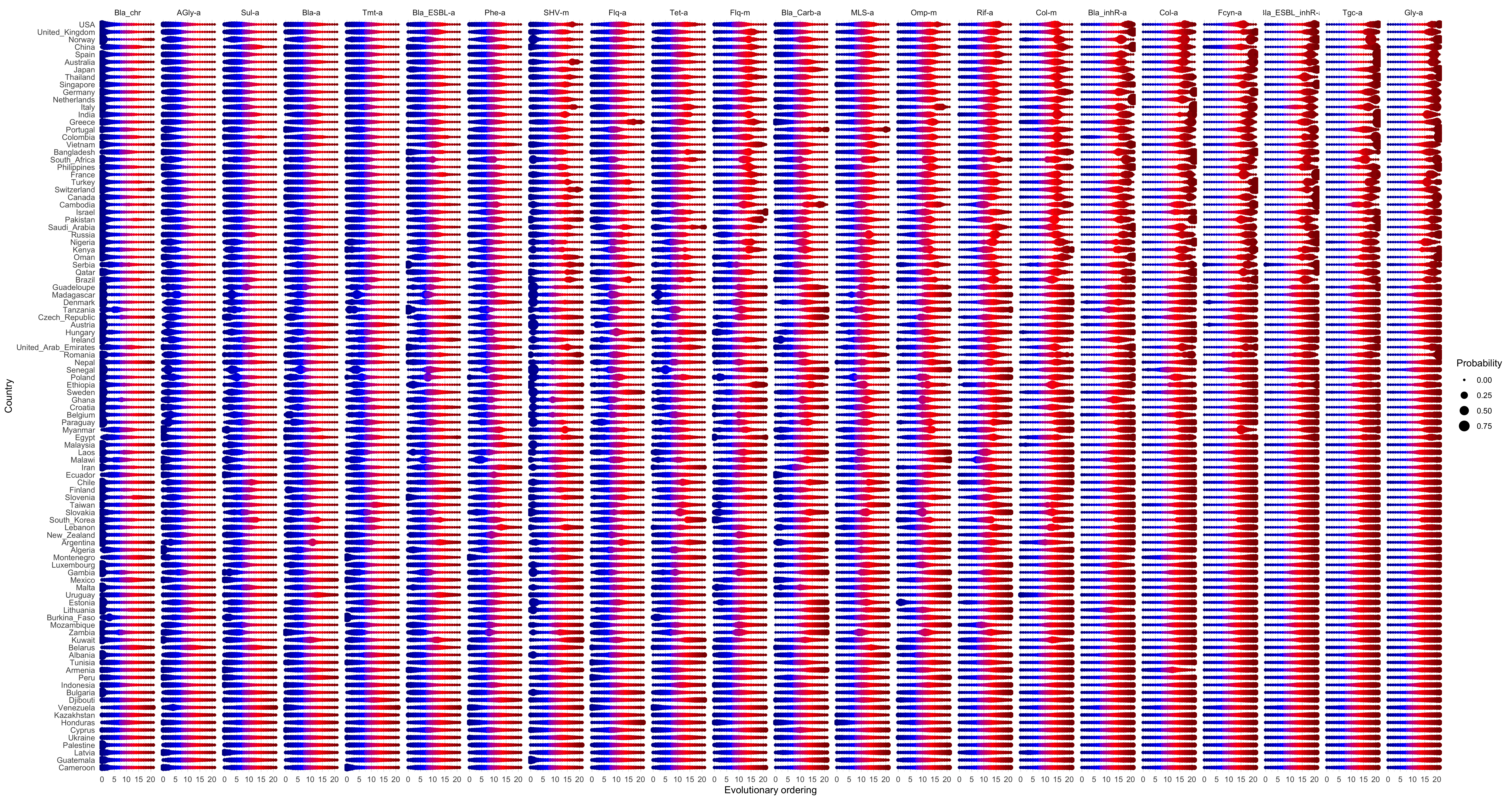

Supplement: S2 Fig — This “global roadmap” summarizes “bubble plots” describing inferred evolutionary dynamics of KpAMR characters in different countries. Each row is a country, ordered vertically by descending number of Kp samples. Each column is a KpAMR character; the horizontal axis within columns gives evolutionary orderings (from early to late). The size of a point gives the probability that that character is acquired at that ordering in that country. For example, across the vast majority of datasets (including the top row, USA, for example), Bla_chr has a high probability of early acquisition and Gly_acquired has a high probability of late acquisition. In Cameroon, all characters have similar inferred evolutionary orderings, reflecting the limited data available to make more precise statements. Character names given in Fig 1 caption. The data and code underlying this figure can be found at https://doi.org/10.5281/zenodo.20408311. (TIF) [file pbio.3003848.s002.tif]

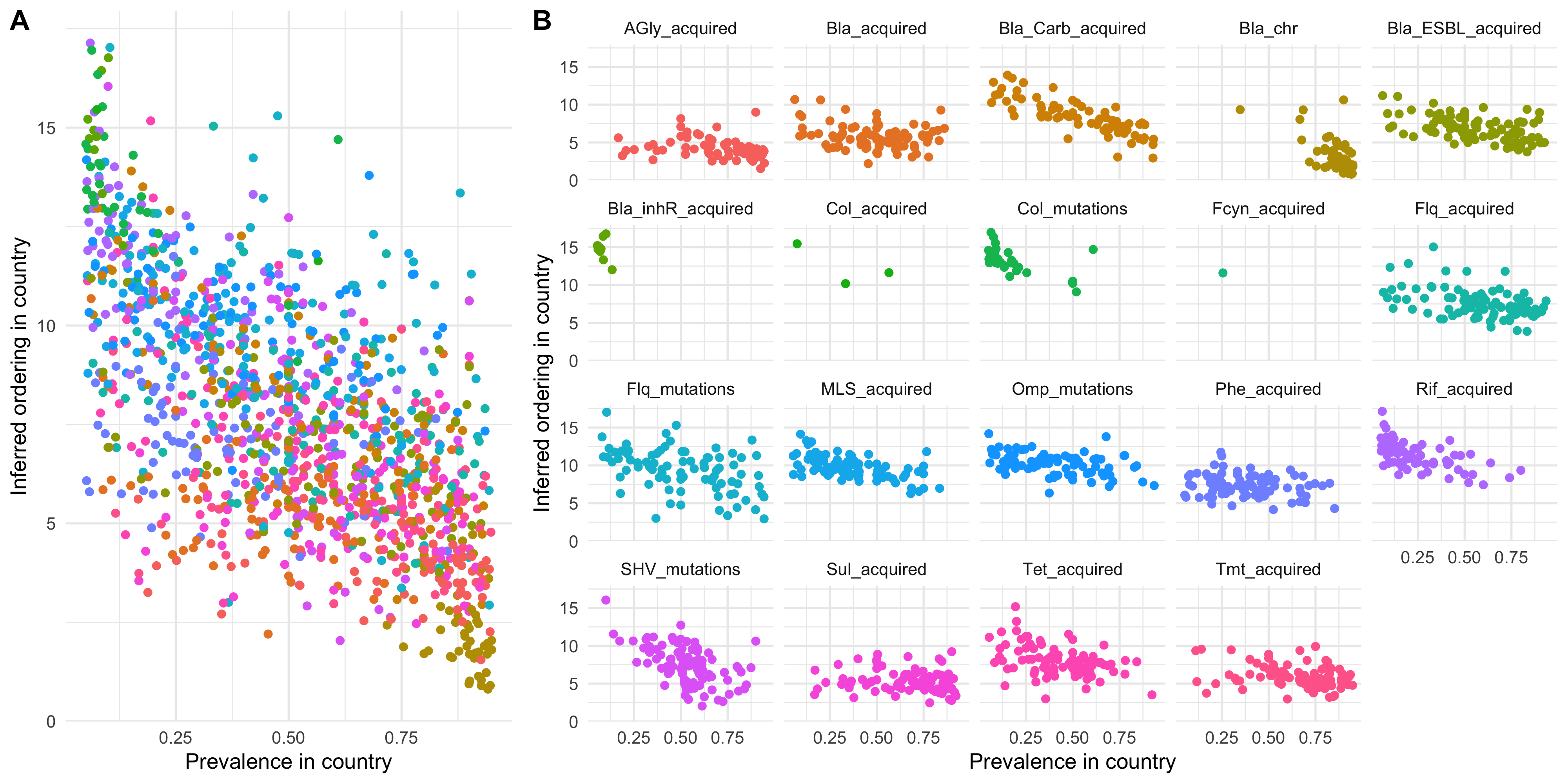

Supplement: S3 Fig — (A) All KpAMR characters with a prevalence >5% and <95% together (R2 = 0.41). (B) Individual KpAMR characters. The data and code underlying this figure can be found at https://doi.org/10.5281/zenodo.20408311. (TIF) [file pbio.3003848.s003.tif]

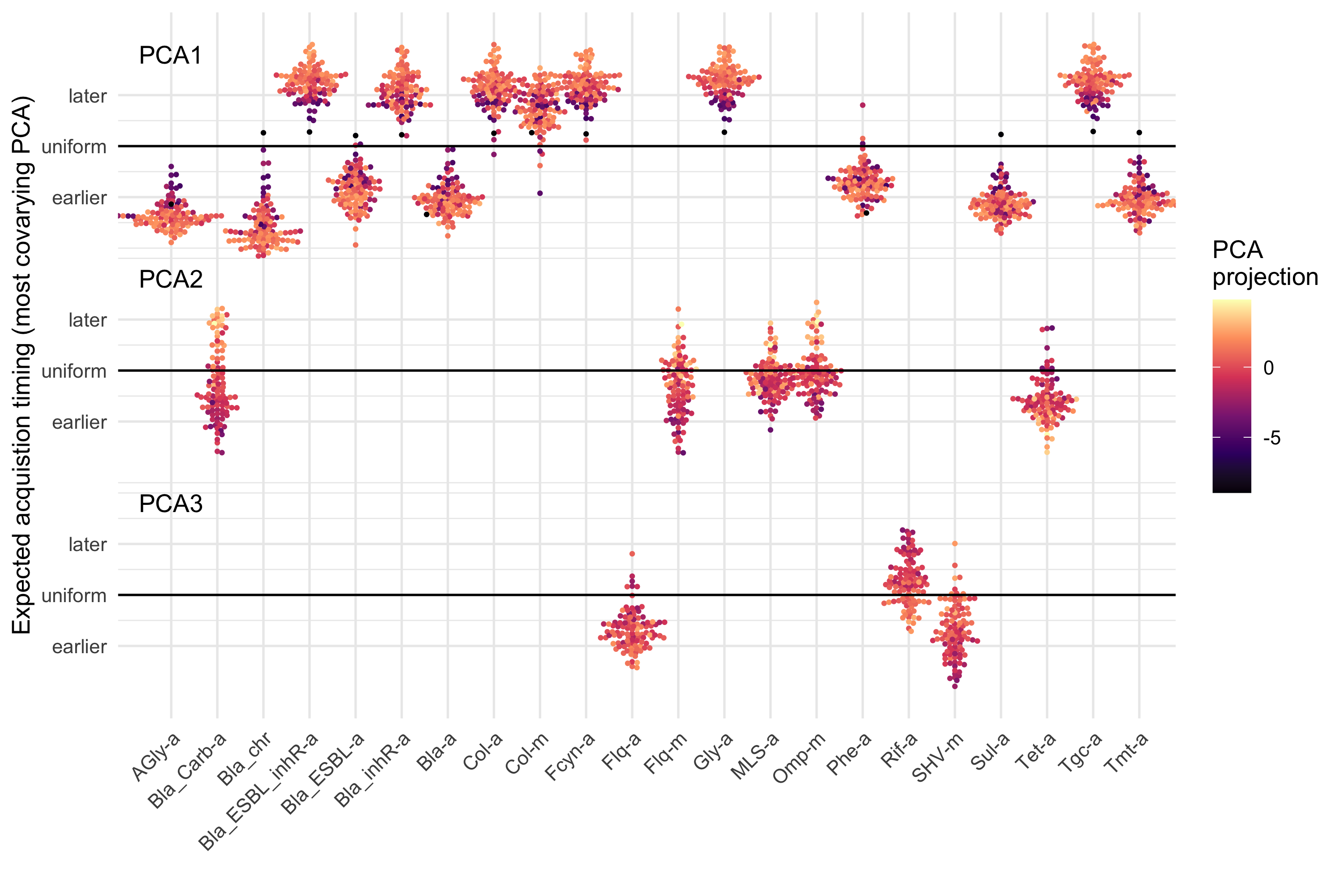

Supplement: S5 Fig — Connected to Fig 3. KpAMR characters (horizontal axis) are plotted by their expected acquisition ordering (vertical axis) for each country (points). The country’s projection on PCA1, PCA2, and PCA3, for those characters that most strongly covary with each PCA axis, are given by the color of a point. As in Fig 3, those characters that covary with PCA1 form a consistent spectrum linked to precision: low values of PCA1 correspond to less precise, more uniform timing, while higher values lead to more precise earlier or later timings. Characters covarying with the other PCAs display wider ranges connected to PCA1-independent variability. Character names given in Fig 1 caption. The data and code underlying this figure can be found at https://doi.org/10.5281/zenodo.20408311. (TIF) [file pbio.3003848.s005.tif]

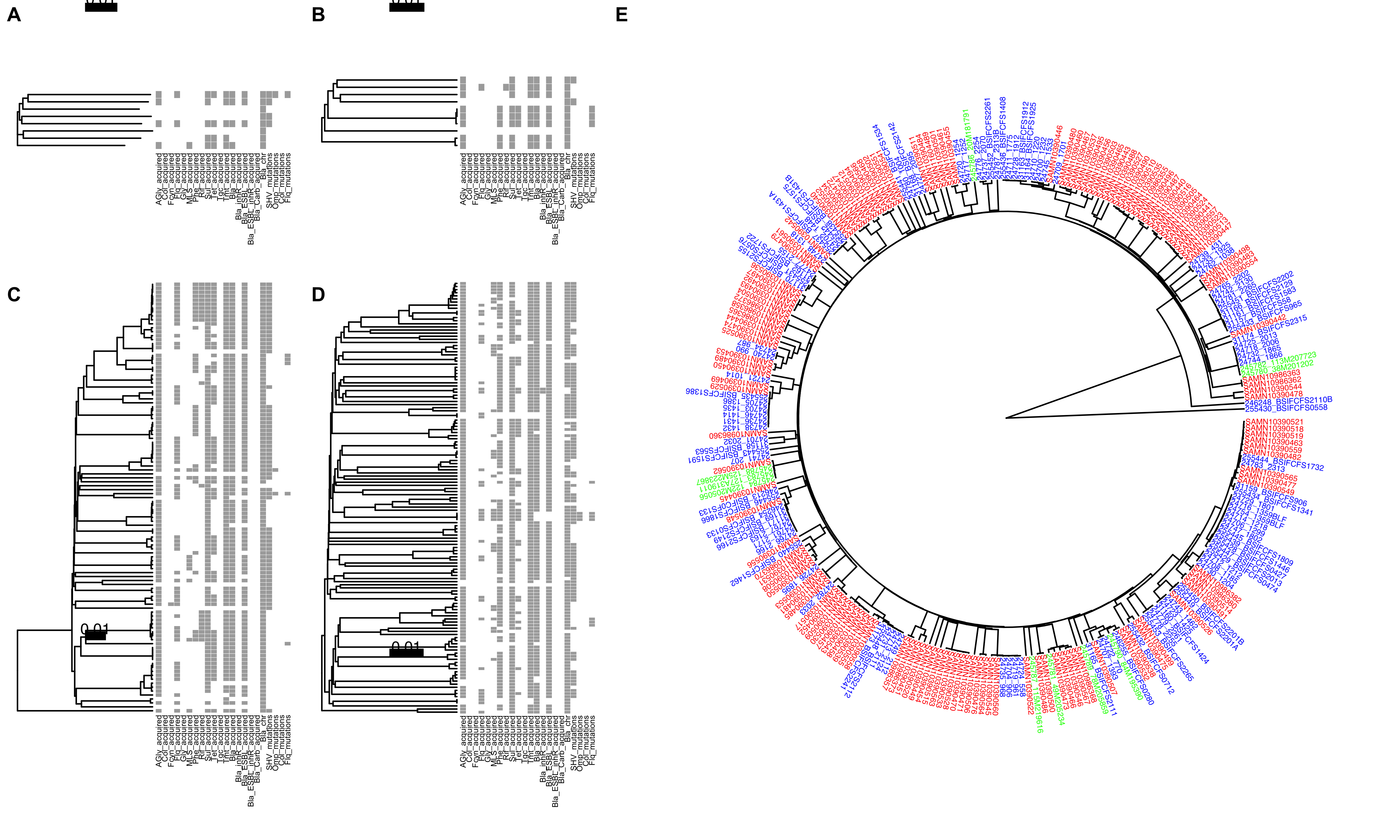

Supplement: S6 Fig — Phylogenies and KpAMR profiles from new datasets: (A) Zanzibar 2015−6. (B) Tanzania 2001−2. (C) Tanzania 2017−8. (D) Pathogenwatch Tanzania. (E) Circular projection of the phylogeny of Tanzanian isolates in Fig 5A. Colors: green, 2001−2; blue, 2017−8; red, existing Pathogenwatch data. The data and code underlying this figure can be found at https://doi.org/10.5281/zenodo.20408311. (TIF) [file pbio.3003848.s006.tif]

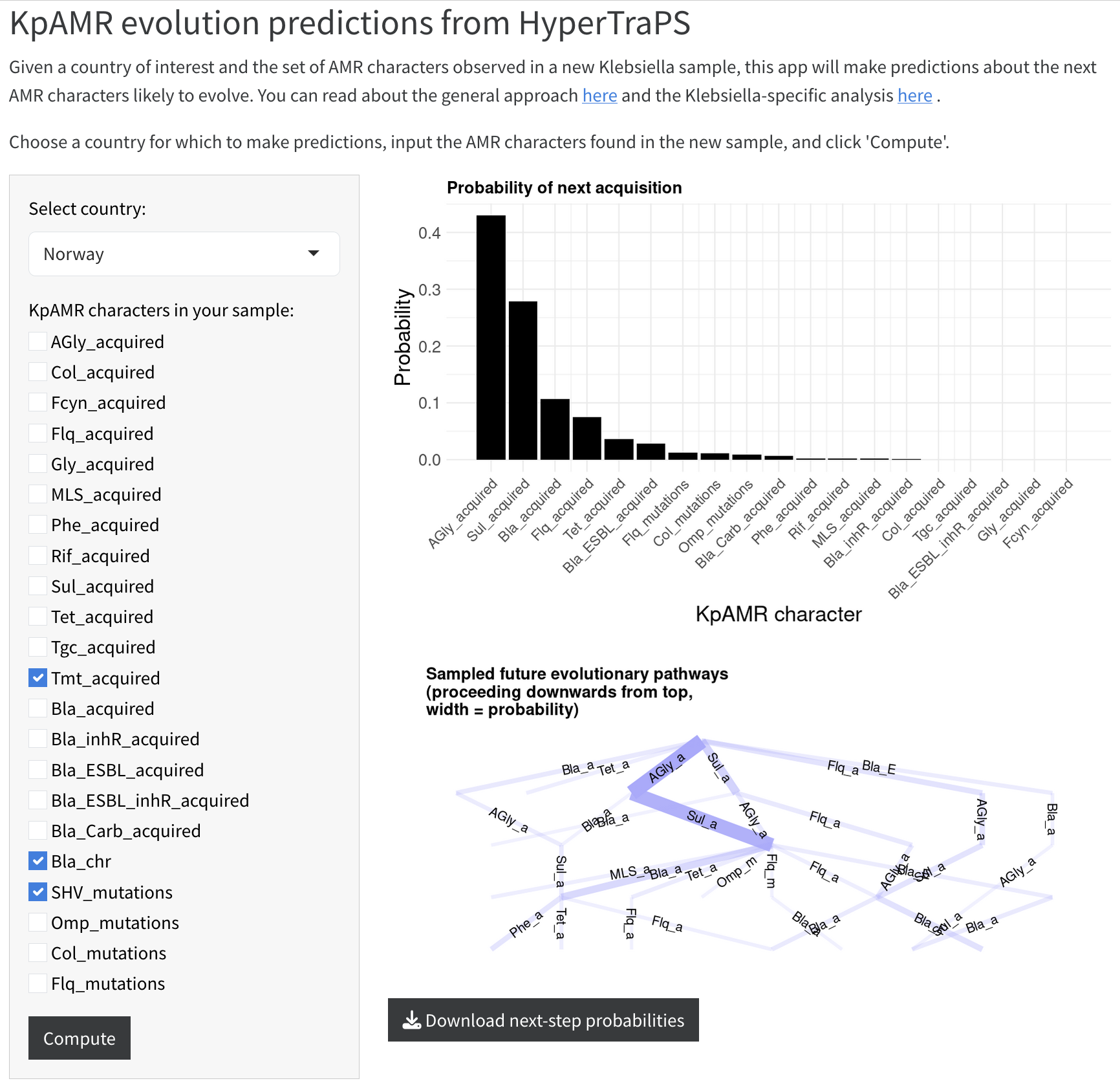

Supplement: S7 Fig — A country of interest is specified, and the KpAMR profile of a sample given by specifying presence or absence of each of our characters. The graphical outputs then give the inferred probabilities of each next possible character acquisition (which can be downloaded in CSV format), as well as a transition graph describing the likely subsequent pathways for up to five acquisitions in the future. Available at https://stochasticbiology.shinyapps.io/amr-predict/. (TIF) [file pbio.3003848.s007.tif]
