# Supplementary material for: Evolutionary inference reveals global natural histories and predicted pathways of antimicrobial resistance in Klebsiella pneumoniae
Source: PLoS Biol. 2026 Jun 12;24(6):e3003848. doi: 10.1371/journal.pbio.3003848 (PMC13278573; doi:10.1371/journal.pbio.3003848)
Supplement: Attachment — Submitted filename: pb-reviewer-response.pdf [file pbio.3003848.s010.pdf]

Reviewer #1 (Joachim Krug):

The scope and significance of the work has been well described in the previous reports. As far as I can see, in the revision the authors have fully addressed the concerns raised by the previous reviewers, by adding material to the manuscript and the supplement, as well as through a new in-depth investigation of the key problem of incorporating reversibility of evolutionary pathways in the inference (which here corresponds to the possibility of acquired AMR features being lost along a trajectory), see Johnston 2026. The detailed engagement with the limitations of the HyperTraPS approach caused by its restriction to irreversible acquisition trajectories has the somewhat unfortunate consequence of weakening the conclusions that can be drawn with confidence, in particular with regard to possible interactions between resistance features that could guide the development of combination therapies. Nevertheless, the study remains an impressive demonstration of the state of the art in the field, and I recommend publication in PLoS Biology after the following (additional) comments and suggestions have been addressed.

Many thanks for your time and energy on our manuscript – we are very grateful for this perspective and for the comments below!

MAJOR ISSUES

1. While the distinction between reversible and irreversible acquisition pathways is discussed in detail in the revised manuscript, it may not become clear to the reader why it is computationally difficult to incorporate reversibility. This is well explained in the new manuscript by Johnston 2026 - it is just a matter of the explosion in the number of possible pathways - and I suggest to include a similar discussion here. To provide some context, it may be helpful to refer to the seminal work of Weinreich and collaborators on trajectories towards high resistance in the TEM-1 beta-lactamase (Science 2006) and the follow-up work by De Pristo et al. (MBE 2007). Weinreich et al. found that out of the 5!=120 possible irreversible trajectories along which 5 mutations in TEM-1 could be acquired only 18 were 'selectively accessible' (= monotonically increasing in MIC). De Pristo et al. expanded the analysis to irreversible trajectories, finding an enormous increase of possible paths (from 120 to 18,651,552,840) but only a moderate increase in the number of accessible ones (from 18 to 27), because very long trajectories are unlikely to be accessible. Weinreichs problem is different from the present one in that in his case trajectories cannot revisit the same configuration (and therefore the number of pathways is finite even when reversions are included), but in practice this distinction hardly matters (in fact in the extensive theoretical work on this problem the constraint that accessible paths are self-avoiding can be ignored asymptotically for large hypercubes, see Schmiegelt and Krug (J. Math. Biol. 2023) for a rigorous treatment and Krug and Oros (Journal of Statistical Mechanics: Theory and Experiment 2024, Section 3) for a review).

Thanks for suggesting an engagement with this literature. We have expanded our discussion of this point in Supp Text 1:

The fundamental reason we use an irreversible model is computational tractability. If reversible transitions are allowed, a potentially infinite set of evolutionary pathways (involving repeated acquisition and loss of characters) can connect any two states, complicating the computation of these pathways and their interpretation. In practise – including in AMR systems – only a limited number of the vast space of possible pathways play important roles in evolutionary dynamics (DePristo et al., 2007; Weinreich et al., 2006), and powerful results have been

demonstrated for accessibility of pathways sets on hypercubic evolution landscapes (Krug & Oros, 2024; Schmiegelt & Krug, 2023).

And provided a pointer and brief description in the paragraph linking to this text:

This irreversibility picture reduces a potentially vast (or infinite) intractable set of possible evolutionary pathways to a tractable set (Supp. Text 1; (DePristo et al., 2007; Weinreich et al., 2006)).

2. An important change compared to the previous version of the manuscript is the choice of null model in the assessment of the predictive potential of the method in Figure 5. The new panel 5B shows that the prediction of the HyperTraPS is almost indistinguishable from that of a simple null model based only on the prevalence of traits. I have difficulty interpreting this figure and linking it to the statement in the text that the trained model outperforms the null model in 18% of cases (lines 359/360). In terms of predicting the exact rank, it seems from Fig. 5B that the two models perform equally well, getting it right in about 50% of cases. How is the full information in the histogram in Fig. 5B used to quantify the overall performance?

The 18% figure comes from the number of cases in Fig 5B where the HyperTraPS model assigns a lower rank (i.e. closer to the "true" rank 1) than the model using prevalence proportions alone. This is the case for 6/34 of the instances comprising the histogram – including, for example, instances where "HyperTraPS" assigns ranks 3/4 and "Prevalence" assigns ranks 7/12. There was one case where "Prevalence" outperformed "HyperTraPS" (1/34 = 3%); apologies for missing this earlier. We have edited the descriptive sentence in the results to clarify these figures.

MINOR ISSUES

3. The abbreviation GBD (= Global Burden of Disease) is used throughout the paper but not defined.

4. line 231: 19th century -> 20th century

5. line 336: remove 'and'

Thanks for catching these (rather embarassing) slips – we have edited each of them.
