# Supplementary material for: Evolutionary inference reveals global natural histories and predicted pathways of antimicrobial resistance in Klebsiella pneumoniae
Source: PLoS Biol. 2026 Jun 12;24(6):e3003848. doi: 10.1371/journal.pbio.3003848 (PMC13278573; doi:10.1371/journal.pbio.3003848)
Supplement: Peer review history

# Editor report: Decision Letter 0

**DOI:** 10.1371/journal.pbio.3003848.r001

**Authors:** Melissa Vazquez Hernandez

1 Apr 2026

Dear Dr Johnston,

Thank you for submitting your manuscript entitled "A natural history of AMR in Klebsiella pneumoniae: Global diversity, predictors, and predictions of evolutionary pathways" for consideration as a Research Article by PLOS Biology.

Your manuscript has now been evaluated by the PLOS Biology editorial staff as well as by an academic editor with relevant expertise and I am writing to let you know that we would like to send your submission out for external peer review.

However, before we can send your manuscript to reviewers, we need you to complete your submission by providing the metadata that is required for full assessment. To this end, please login to Editorial Manager where you will find the paper in the 'Submissions Needing Revisions' folder on your homepage. Please click 'Revise Submission' from the Action Links and complete all additional questions in the submission questionnaire.

Once your full submission is complete, your paper will undergo a series of checks in preparation for peer review. After your manuscript has passed the checks it will be sent out for review. To provide the metadata for your submission, please Login to Editorial Manager (https://www.editorialmanager.com/pbiology) within two working days, i.e. by Apr 03 2026 11:59PM.

If your manuscript has been previously peer-reviewed at another journal, PLOS Biology is willing to work with those reviews in order to avoid re-starting the process. Submission of the previous reviews is entirely optional and our ability to use them effectively will depend on the willingness of the previous journal to confirm the content of the reports and share the reviewer identities. Please note that we reserve the right to invite additional reviewers if we consider that additional/independent reviewers are needed, although we aim to avoid this as far as possible. In our experience, working with previous reviews does save time.

If you would like us to consider previous reviewer reports, please edit your cover letter to let us know and include the name of the journal where the work was previously considered and the manuscript ID it was given. In addition, please upload a response to the reviews as a 'Prior Peer Review' file type, which should include the reports in full and a point-by-point reply detailing how you have or plan to address the reviewers' concerns.

During the process of completing your manuscript submission, you will be invited to opt-in to posting your pre-review manuscript as a bioRxiv preprint. Visit http://journals.plos.org/plosbiology/s/preprints for full details. If you consent to posting your current manuscript as a preprint, please upload a single Preprint PDF.

Feel free to email us at plosbiology@plos.org if you have any queries relating to your submission.

Kind regards,

Melissa

Melissa Vazquez Hernandez, Ph.D.

Associate Editor

PLOS Biology

mvazquezhernandez@plos.org

# Peer review: Decision Letter 1

**DOI:** 10.1371/journal.pbio.3003848.r002

**Authors:** Melissa Vazquez Hernandez

28 Apr 2026

Dear Iain,

Thank you for your patience while we considered your revised manuscript "A natural history of AMR in Klebsiella pneumoniae: Global diversity, predictors, and predictions of evolutionary pathways" for consideration as a Research Article at PLOS Biology under Portable Peer Review. Your revised study has now been evaluated by the PLOS Biology editors, the Academic Editor and one additional reviewer (Joachim Krug).

In light of the reviews, which you will find at the end of this email, we would like to invite you to revise your manuscript to address several points raised during evaluation. As you will see below, the additional reviewer is overall positive about the quality and relevance of the work and acknowledges that the previous concerns brought at the previous journal have been carefully addressed, particularly with the expanded treatment of reversibility. However, some important issues remain that require clarification before further consideration. In particular, our reviewer asks that you more clearly explain the computational challenges associated with incorporating reversibility into the modeling framework, to better guide readers through this conceptual limitation. In addition, s/he raises concerns about the interpretation of the predictive analysis presented in Figure 5, noting that the performance of the HyperTraPS model appears comparable to a simple prevalence-based null model. We therefore ask that you clarify how predictive performance is quantified and revise the text to more accurately reflect the strength of this result. More generally, we encourage you to carefully temper conclusions regarding inferred interactions between resistance features, given the acknowledged limitations of the approach.

In addition to these revisions, you may need to complete some formatting changes, which you will receive in a follow up email. A member of our team will be in touch with a set of requests shortly. If you do not receive a separate email within a few days, please assume that checks have been completed, and no additional changes are required.

We expect to receive your revised manuscript within 1 month. Please email us (plosbiology@plos.org) if you have any questions or concerns, or would like to request an extension.

At this stage, your manuscript remains formally under active consideration at our journal; please notify us by email if you do not intend to submit a revision so that we withdraw the manuscript.

**IMPORTANT - SUBMITTING YOUR REVISION**

Your revisions should address the specific points made by each reviewer. Please submit the following files along with your revised manuscript:

1. A 'Response to Reviewers' file - this should detail your responses to the editorial requests, present a point-by-point response to all of the reviewers' comments, and indicate the changes made to the manuscript.

*NOTE: In your point-by-point response to the reviewers, please provide the full context of each review. Do not selectively quote paragraphs or sentences to reply to. The entire set of reviewer comments should be present in full and each specific point should be responded to individually.

You should also cite any additional relevant literature that has been published since the original submission and mention any additional citations in your response.

2. In addition to a clean copy of the manuscript, please also upload a 'track-changes' version of your manuscript that specifies the edits made. This should be uploaded as a "Revised Article with Changes Highlighted " file type.

*Resubmission Checklist*

When you are ready to resubmit your revised manuscript, please refer to this resubmission checklist: https://plos.io/Biology_Checklist

To submit a revised version of your manuscript, please go to https://www.editorialmanager.com/pbiology/ and log in as an Author. Click the link labelled 'Submissions Needing Revision' where you will find your submission record.

Please make sure to read the following important policies and guidelines while preparing your revision:

*Published Peer Review*

Please note while forming your response, if your article is accepted, you may have the opportunity to make the peer review history publicly available. The record will include editor decision letters (with reviews) and your responses to reviewer comments. If eligible, we will contact you to opt in or out. Please see here for more details:

https://blogs.plos.org/plos/2019/05/plos-journals-now-open-for-published-peer-review/

*PLOS Data Policy*

Please note that as a condition of publication PLOS' data policy (http://journals.plos.org/plosbiology/s/data-availability) requires that you make available all data used to draw the conclusions arrived at in your manuscript. If you have not already done so, you must include any data used in your manuscript either in appropriate repositories, within the body of the manuscript, or as supporting information (N.B. this includes any numerical values that were used to generate graphs, histograms etc.). For an example see here: http://www.plosbiology.org/article/info%3Adoi%2F10.1371%2Fjournal.pbio.1001908#s5

*Blot and Gel Data Policy*

We require the original, uncropped and minimally adjusted images supporting all blot and gel results reported in an article's figures or Supporting Information files. We will require these files before a manuscript can be accepted so please prepare them now, if you have not already uploaded them. Please carefully read our guidelines for how to prepare and upload this data: https://journals.plos.org/plosbiology/s/figures#loc-blot-and-gel-reporting-requirements

*Protocols deposition*

To enhance the reproducibility of your results, we recommend that if applicable you deposit your laboratory protocols in protocols.io, where a protocol can be assigned its own identifier (DOI) such that it can be cited independently in the future. Additionally, PLOS ONE offers an option for publishing peer-reviewed Lab Protocol articles, which describe protocols hosted on protocols.io. Read more information on sharing protocols at https://plos.org/protocols?utm_medium=editorial-email&utm_source=authorletters&utm_campaign=protocols

Thank you again for your submission to our journal. We hope that our editorial process has been constructive thus far, and we welcome your feedback at any time. Please don't hesitate to contact us if you have any questions or comments.

Sincerely,

Melissa

Melissa Vazquez Hernandez, Ph.D.

Associate Editor

PLOS Biology

mvazquezhernandez@plos.org

----------------------------------------------------------------

Reviewer #1 (Joachim Krug):

The scope and significance of the work has been well described in the previous reports. As far as I can see, in the revision the authors have fully addressed the concerns raised by the previous reviewers, by adding material to the manuscript and the supplement, as well as through a new in-depth investigation of the key problem of incorporating reversibility of evolutionary pathways in the inference (which here corresponds to the possibility of acquired AMR features being lost along a trajectory), see Johnston 2026. The detailed engagement with the limitations of the HyperTraPS approach caused by its restriction to irreversible acquisition trajectories has the somewhat unfortunate consequence of weakening the conclusions that can be drawn with confidence, in particular with regard to possible interactions between resistance features that could guide the development of combination therapies. Nevertheless, the study remains an impressive demonstration of the state of the art in the field, and I recommend publication in PLoS Biology after the following (additional) comments and suggestions have been addressed.

MAJOR ISSUES

1. While the distinction between reversible and irreversible acquisition pathways is discussed in detail in the revised manuscript, it may not become clear to the reader why it is computationally difficult to incorporate reversibility. This is well explained in the new manuscript by Johnston 2026 - it is just a matter of the explosion in the number of possible pathways - and I suggest to include a similar discussion here. To provide some context, it may be helpful to refer to the seminal work of Weinreich and collaborators on trajectories towards high resistance in the TEM-1 beta-lactamase (Science 2006) and the follow-up work by De Pristo et al. (MBE 2007). Weinreich et al. found that out of the 5!=120 possible irreversible trajectories along which 5 mutations in TEM-1 could be acquired only 18 were 'selectively accessible' (= monotonically increasing in MIC). De Pristo et al. expanded the analysis to irreversible trajectories, finding an enormous increase of possible paths (from 120 to 18,651,552,840) but only a moderate increase in the number of accessible ones (from 18 to 27), because very long trajectories are unlikely to be accessible. Weinreichs problem is different from the present one in that in his case trajectories cannot revisit the same configuration (and therefore the number of pathways is finite even when reversions are included), but in practice this distinction hardly matters (in fact in the extensive theoretical work on this problem the constraint that accessible paths are self-avoiding can be ignored asymptotically for large hypercubes, see Schmiegelt and Krug (J. Math. Biol. 2023) for a rigorous treatment and Krug and Oros (Journal of Statistical Mechanics: Theory and Experiment 2024, Section 3) for a review).

2. An important change compared to the previous version of the manuscript is the choice of null model in the assessment of the predictive potential of the method in Figure 5. The new panel 5B shows that the prediction of the HyperTraPS is almost indistinguishable from that of a simple null model based only on the prevalence of traits. I have difficulty interpreting this figure and linking it to the statement in the text that the trained model outperforms the null model in 18% of cases (lines 359/360). In terms of predicting the exact rank, it seems from Fig. 5B that the two models perform equally well, getting it right in about 50% of cases. How is the full information in the histogram in Fig. 5B used to quantify the overall performance?

MINOR ISSUES

3. The abbreviation GBD (= Global Burden of Disease) is used throughout the paper but not defined.

4. line 231: 19th century -> 20th century

5. line 336: remove 'and'

# Author comment: Author response to Decision Letter 2

**DOI:** 10.1371/journal.pbio.3003848.r003

7 May 2026

Attachment Submitted filename: pb-reviewer-response.pdf

# Peer review: Decision Letter 2

**DOI:** 10.1371/journal.pbio.3003848.r004

**Authors:** Melissa Vazquez Hernandez

25 May 2026

Dear Iain,

Thank you for your patience while we considered your revised manuscript "A natural history of AMR in Klebsiella pneumoniae: Global diversity, predictors, and predictions of evolutionary pathways" for publication as a Research Article at PLOS Biology. This revised version of your manuscript has been evaluated by the PLOS Biology editors, the Academic Editor and the original reviewer.

Based on the review, we are likely to accept this manuscript for publication, provided you satisfactorily address the remaining editorial points. Please also make sure to address the following data and other policy-related requests.

1) We routinely suggest changes to titles to ensure maximum accessibility for a broad, non-specialist readership, and to ensure they reflect the contents of the paper. In this case, we would suggest a minor edit to the title, as follows. Please ensure you change both the manuscript file and the online submission system, as they need to match for final acceptance:

"Evolutionary modeling reveals global routes to antimicrobial resistance in Klebsiella pneumoniae"

2) Please add the weblink of the funding agencies in the Financial Disclosure statement in the manuscript details during submission.

3) We do not have a word limit. Please move the supplementary text, together with the references, to the main text which can provide the readers an easier access to all information.

4) You may be aware of the PLOS Data Policy, which requires that all data be made available without restriction: http://journals.plos.org/plosbiology/s/data-availability. For more information, please also see this editorial: http://dx.doi.org/10.1371/journal.pbio.1001797

Please supply the numerical values either in the a supplementary file or as a permanent DOI’d deposition for the following figures:

Figure 1ABC, 2AC, 3ABC, 4A-D, 5BC, S1ABC, S2, S3AB, S4A, S5

NOTE: the numerical data provided should include all replicates AND the way in which the plotted mean and errors were derived (it should not present only the mean/average values).

*If the figure were done using r, or any code, please provide the code necessary to replicate the figures.

5) Please cite the location of the data clearly in all relevant main and supplementary Figure legends, e.g. “The data underlying this Figure can be found in S1 Data” or “The data underlying this Figure can be found in https://doi.org/10.5281/zenodo.XXXXX”

6) Please provide the tree files for the phylogenetic trees in Figures .5A, S1AB, S6A-E Please make sure all relevant figures have scale bars.

7) Please ensure that your Data Statement in the submission system accurately describes where your data can be found and is in final format, as it will be published as written there

8) Thank you for providing the underlying code in GitHub. However, because Github depositions can be readily changed or deleted, please make a permanent DOI’d copy (e.g. in Zenodo) and provide this URL in the manuscript and Data Availability Statement.

As you address these items, please take this last chance to review your reference list to ensure that it is complete and correct. If you have cited papers that have been retracted, please include the rationale for doing so in the manuscript text, or remove these references and replace them with relevant current references. Any changes to the reference list should be mentioned in the cover letter that accompanies your revised manuscript.

In addition to these revisions, you may need to complete some formatting changes, which you will receive in a follow up email. A member of our team will be in touch with a set of requests shortly. If you do not receive a separate email within a few days, please assume that checks have been completed, and no additional changes are required.

We expect to receive your revised manuscript within two weeks.

To submit your revision, please go to https://www.editorialmanager.com/pbiology/ and log in as an Author. Click the link labelled 'Submissions Needing Revision' to find your submission record. Your revised submission must include the following:

- a cover letter that should detail your responses to any editorial requests, if applicable, and whether changes have been made to the reference list

- a Response to Reviewers file that provides a detailed response to the reviewers' comments (if applicable, if not applicable please do not delete your existing 'Response to Reviewers' file.)

- a track-changes file indicating any changes that you have made to the manuscript.

NOTE: If Supporting Information files are included with your article, note that these are not copyedited and will be published as they are submitted. Please ensure that these files are legible and of high quality (at least 300 dpi) in an easily accessible file format. For this reason, please be aware that any references listed in an SI file will not be indexed. For more information, see our Supporting Information guidelines:

https://journals.plos.org/plosbiology/s/supporting-information

*Published Peer Review History*

Please note that you may have the opportunity to make the peer review history publicly available. The record will include editor decision letters (with reviews) and your responses to reviewer comments. If eligible, we will contact you to opt in or out. Please see here for more details:

https://plos.org/published-peer-review-history/

*Press*

Should you, your institution's press office or the journal office choose to press release your paper, please ensure you have opted out of Early Article Posting on the submission form. We ask that you notify us as soon as possible if you or your institution is planning to press release the article.

*Protocols deposition*

To enhance the reproducibility of your results, we recommend that if applicable you deposit your laboratory protocols in protocols.io, where a protocol can be assigned its own identifier (DOI) such that it can be cited independently in the future. Additionally, PLOS ONE offers an option for publishing peer-reviewed Lab Protocol articles, which describe protocols hosted on protocols.io. Read more information on sharing protocols at https://plos.org/protocols?utm_medium=editorial-email&utm_source=authorletters&utm_campaign=protocols

Please do not hesitate to contact me should you have any questions.

Sincerely,

Melissa

Melissa Vazquez Hernandez, Ph.D.

Associate Editor

mvazquezhernandez@plos.org

PLOS Biology

------------------------------------------------------------------------

REVIEWERS' COMMENTS

------------------------------------------------------------------------

Reviewer #1 (Joachim Krug):

My previous comments have been fully addressed in the revision.

# Editor report: Decision Letter 3

**DOI:** 10.1371/journal.pbio.3003848.r005

**Authors:** Melissa Vazquez Hernandez

28 May 2026

Dear Iain

Thank you for the submission of your revised Research Article "Evolutionary inference reveals global natural histories and predicted pathways of antimicrobial resistance in Klebsiella pneumoniae" for publication in PLOS Biology, which came in as Portable Peer Review. On behalf of my colleagues and the Academic Editor, J. Arjan G. M. de Visser, I am pleased to say that we can in principle accept your manuscript for publication, provided you address any remaining formatting and reporting issues. These will be detailed in an email you should receive within 2-3 business days from our colleagues in the journal operations team; no action is required from you until then. Please note that we will not be able to formally accept your manuscript and schedule it for publication until you have completed any requested changes.

Please take a minute to log into Editorial Manager at http://www.editorialmanager.com/pbiology/, click the "Update My Information" link at the top of the page, and update your user information to ensure an efficient production process.

PRESS

We frequently collaborate with press offices. If your institution or institutions have a press office, please notify them about your upcoming paper at this point, to enable them to help maximise its impact. If the press office is planning to promote your findings, we would be grateful if they could coordinate with biologypress@plos.org. If you have previously opted in to the early version process, we ask that you notify us immediately of any press plans so that we may opt out on your behalf.

We also ask that you take this opportunity to read our Embargo Policy regarding the discussion, promotion and media coverage of work that is yet to be published by PLOS. As your manuscript is not yet published, it is bound by the conditions of our Embargo Policy. Please be aware that this policy is in place both to ensure that any press coverage of your article is fully substantiated and to provide a direct link between such coverage and the published work. For full details of our Embargo Policy, please visit http://www.plos.org/about/media-inquiries/embargo-policy/.

Thank you again for choosing PLOS Biology for publication and supporting Open Access publishing. We look forward to publishing your study.

Sincerely,

Melissa

Melissa Vazquez Hernandez, Ph.D., Ph.D.

Associate Editor

PLOS Biology

mvazquezhernandez@plos.org
